# Leprosy and the Adaptation of Human Toll-Like Receptor 1

**DOI:** 10.1371/journal.ppat.1000979

**Published:** 2010-07-01

**Authors:** Sunny H. Wong, Sailesh Gochhait, Dheeraj Malhotra, Fredrik H. Pettersson, Yik Y. Teo, Chiea C. Khor, Anna Rautanen, Stephen J. Chapman, Tara C. Mills, Amit Srivastava, Aleksey Rudko, Maxim B. Freidin, Valery P. Puzyrev, Shafat Ali, Shweta Aggarwal, Rupali Chopra, Belum S. N. Reddy, Vijay K. Garg, Suchismita Roy, Sarah Meisner, Sunil K. Hazra, Bibhuti Saha, Sian Floyd, Brendan J. Keating, Cecilia Kim, Benjamin P. Fairfax, Julian C. Knight, Philip C. Hill, Richard A. Adegbola, Hakon Hakonarson, Paul E. M. Fine, Ramasamy M. Pitchappan, Rameshwar N. K. Bamezai, Adrian V. S. Hill, Fredrik O. Vannberg

**Affiliations:** 1 Wellcome Trust Centre for Human Genetics, University of Oxford, Oxford, United Kingdom; 2 National Centre of Applied Human Genetics, School of Life Sciences, Jawaharlal Nehru University, New Delhi, India; 3 Host Susceptibility to Infection Program, Singapore Institute for Clinical Sciences, Agency for Science, Technology and Research, Singapore; 4 Research Institute of Medical Genetics, Siberian Branch of Russian Academy of Medical Sciences, Tomsk, Russia; 5 Department of Dermatology and Sexually Transmitted Diseases, Maulana Azad Medical College, Lok Nayak Jai Prakash Hospital, New Delhi, India; 6 Bath Royal United Hospital Trust, Combe Park, Bath, United Kingdom; 7 Calcutta School of Tropical Medicine, Kolkata, India; 8 Infectious Disease Epidemiology Unit, London School of Hygiene and Tropical Medicine, London, United Kingdom; 9 Institute for Translational Medicine and Therapeutics, School of Medicine, University of Pennsylvania, Philadelphia, Pennsylvania, United States of America; 10 Center for Applied Genomics, Abramson Research Center, The Children's Hospital of Philadelphia, Philadelphia, Pennsylvania, United States of America; 11 MRC Laboratories, Fajara, The Gambia; 12 Centre for Excellence in Genomic Sciences, Madurai Kamaraj University, Madurai, India; Johns Hopkins School of Medicine, United States of America

## Abstract

Leprosy is an infectious disease caused by the obligate intracellular pathogen *Mycobacterium leprae* and remains endemic in many parts of the world. Despite several major studies on susceptibility to leprosy, few genomic loci have been replicated independently. We have conducted an association analysis of more than 1,500 individuals from different case-control and family studies, and observed consistent associations between genetic variants in both *TLR1* and the *HLA-DRB1/DQA1* regions with susceptibility to leprosy (*TLR1* I602S, case-control *P* = 5.7×10^−8^, OR = 0.31, 95% CI = 0.20–0.48, and *HLA-DQA1* rs1071630, case-control *P* = 4.9×10^−14^, OR = 0.43, 95% CI = 0.35–0.54). The effect sizes of these associations suggest that *TLR1* and *HLA-DRB1/DQA1* are major susceptibility genes in susceptibility to leprosy. Further population differentiation analysis shows that the *TLR1* locus is extremely differentiated. The protective dysfunctional 602S allele is rare in Africa but expands to become the dominant allele among individuals of European descent. This supports the hypothesis that this locus may be under selection from mycobacteria or other pathogens that are recognized by TLR1 and its co-receptors. These observations provide insight into the long standing host-pathogen relationship between human and mycobacteria and highlight the key role of the TLR pathway in infectious diseases.

## Author Summary

*Mycobacterium leprae* is an obligate intracellular pathogen that causes leprosy, a disease that shares a long history with the human population but which remains endemic in many parts of the world. Despite the fact that the genome of *M. leprae* has been sequenced, our understanding of its pathogenesis and interaction with the human host is limited, in part due to the inability to culture the bacterium *in vitro*. In this gene-centric microarray study, we have genotyped SNPs in over 2,000 genes and identified *TLR1* and *HLA-DRB1/DQA1* as major leprosy susceptibility genes. Studying the geographical distribution of this hypo-functional TLR1 variant demonstrated extreme population differentiation at this locus. These results suggest that leprosy may have contributed to the evolution of this genomic region, and provide insight into the long history of the host-pathogen relationship between humans and *M. leprae*.

## Introduction

Leprosy is a chronic granulomatous disease affecting the skin and peripheral nerves and caused by *Mycobacterium leprae*. Despite its being the first identified pathogen in humans, leprosy remains endemic in central Africa, Southeast Asia and South America with more than 200,000 new cases per year globally. Our understanding of the bacterial pathogenesis and interaction with the human host is limited, because of the inability to culture the bacterium *in vitro*. Nevertheless, twin studies [1], familial clustering [2] and segregation analyses [3] studies have suggested that host genetics play an important role in susceptibility to this infectious disease with the heritability estimated up to 57% [4]. This allows genetic research to help understand the immunity against *M. leprae* and provide insight into the host-pathogen relationship.

Although several genetic loci have been reported to associate with susceptibility to leprosy, candidates showing independent replications are scanty, and the vast majority of the phenotypic variation remains unexplained. To identify the genetic variants affecting susceptibility to leprosy, we conducted a population based case-control association study using a gene-centric 50 K microarray covering variants in 2,092 genes throughout the genome [5], and found *TLR1* and *HLA-DRB1/DQA1* as major determinants of leprosy susceptibility. We also observe a high degree of population differentiation at the *TLR1* gene, suggesting that mycobacterial diseases may have contributed to the evolution of this locus. These observations refine our understanding of the long interaction between human and mycobacteria and suggest that modulation of the TLR1 pathway may be valuable in future treatment of mycobacterial diseases.

## Results

### Primary association analysis

**Figure 1:**
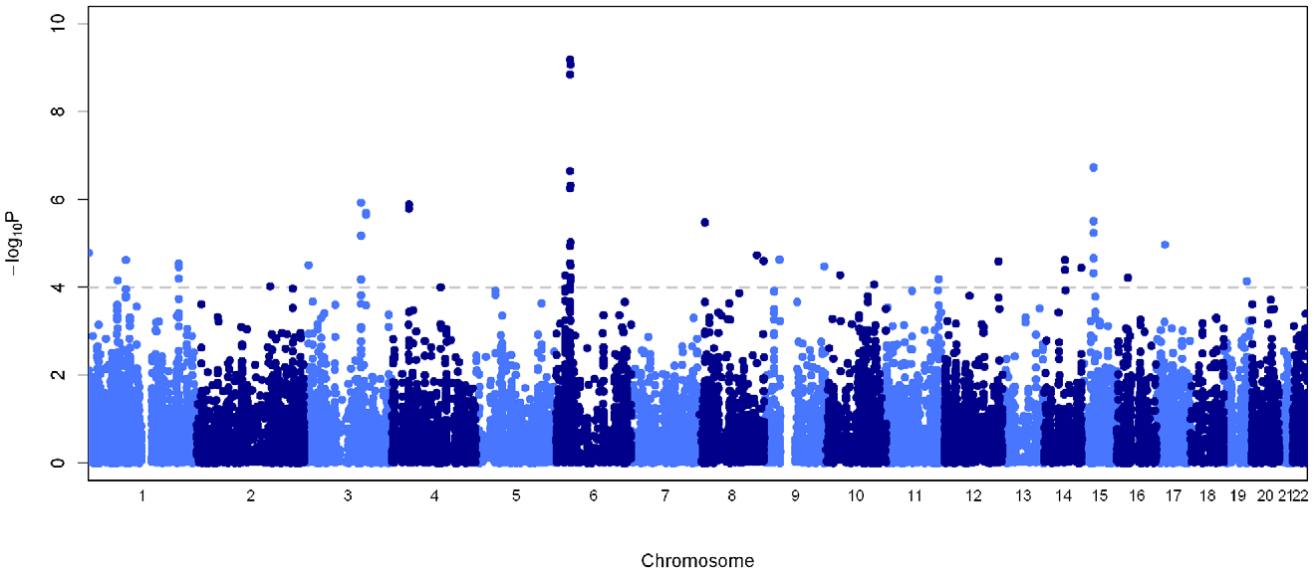
Manhattan plot showing the primary association statistics for the 50 K microarray. Association statistics of the tuberculosis cases versus controls are presented as negative logarithms of the *P*-values. The dotted grey line indicates the threshold *P* = 1.0×10^−4^.

We genotyped 258 leprosy cases and 300 controls recruited from New Delhi, India where the disease is prevalent. Diagnosis of leprosy was made by at least two independent leprologists with standard histopathological examination of affected skin lesions. After quality control filtering, the genotype rate was 99.5% with separate multi-dimensional scaling (MDS) and principal component analysis (PCA) showing minimal population substructure in the 448 individuals (209 cases and 239 controls) carried forward for analysis (**Figures S1** and **S2, Tables S1** and **S2**). The strongest association was observed at the human leukocyte antigen (*HLA*) class II locus at chromosome 6p21 (**Figure 1**), with two SNPs showing genome-wide level of association (rs9270650 *P* = 6.4×10^−10^ and rs1071630 *P* = 8.5×10^−10^, **Table 1** **, Table S3**).

**Table 1:** Association statistics of rs1071630, rs9270650 and rs5743618 in the different cohorts.

*HLA-DRB1/DQA1*									
SNP	Cohort	Cohort Type	Allele	Case Freq	Control Freq	T∶U	OR	95% CI	*P-value*
rs1071630	New Delhi	Case-control	T	68.6%	47.8%	-	2.38	1.80–3.15	8.5×10^−10^
	Kolkata	Case-control	T	71.8%	53.9%	-	2.17	1.53–3.08	1.1×10^−5^
	Kumbakonam	Family	T	-	-	187∶151	1.24	1.00–1.53	0.050
	Combined case-control (Woolf's test *P* = 0.69)						2.30	1.85–2.86	4.9×10^−14^
rs9270650	New Delhi	Case-control	C	42.9%	21.8%	-	2.70	1.93–3.76	6.4×10^−10^
	Kolkata	Case-control	C	48.6%	34.1%	-	1.83	1.28–2.59	7.5×10^−4^
	Kumbakonam	Family	C	-	-	156∶120	1.30	1.03–1.65	0.030
	Combined case-control (Woolf's test *P* = 0.11)						2.24	1.76–2.86	3.1×10^−11^

*TLR1*									
SNP	Cohort	Cohort type	Allele	Case	Control	T∶U	OR	95% CI	*P-value*
rs5743618	New Delhi	Case-control	C	3.8%	13.0%	-	0.27	0.15–0.47	1.3×10^−6^
	Kolkata	Case-control	C	3.6%	8.4%	-	0.40	0.20–0.83	0.012
	Turkey [8]	Case-control	C	26.3%	42.8%	-	0.48	0.29–0.80	0.004
	Kumbakonam	Family	C	-	-	19∶31	0.61	0.35–1.09	0.090
	Combined (Indian case-control, Woolf's test *P* = 0.37)						0.31	0.20–0.48	5.7×10^−8^
	Combined (all case-control, Woolf's test *P* = 0.33)						0.37	0.26–0.51	1.7×10^−9^

The Pearson's χ^2^ allelic test was presented for the association test in the case-control cohorts, and the transmission-disequilibrium-test was used for the association test in the family-based cohort. The data from the Turkey cohort were retrieved from the study by Johnson *et al*.

### Human leukocyte antigen (*HLA*) region

**Figure 2:**
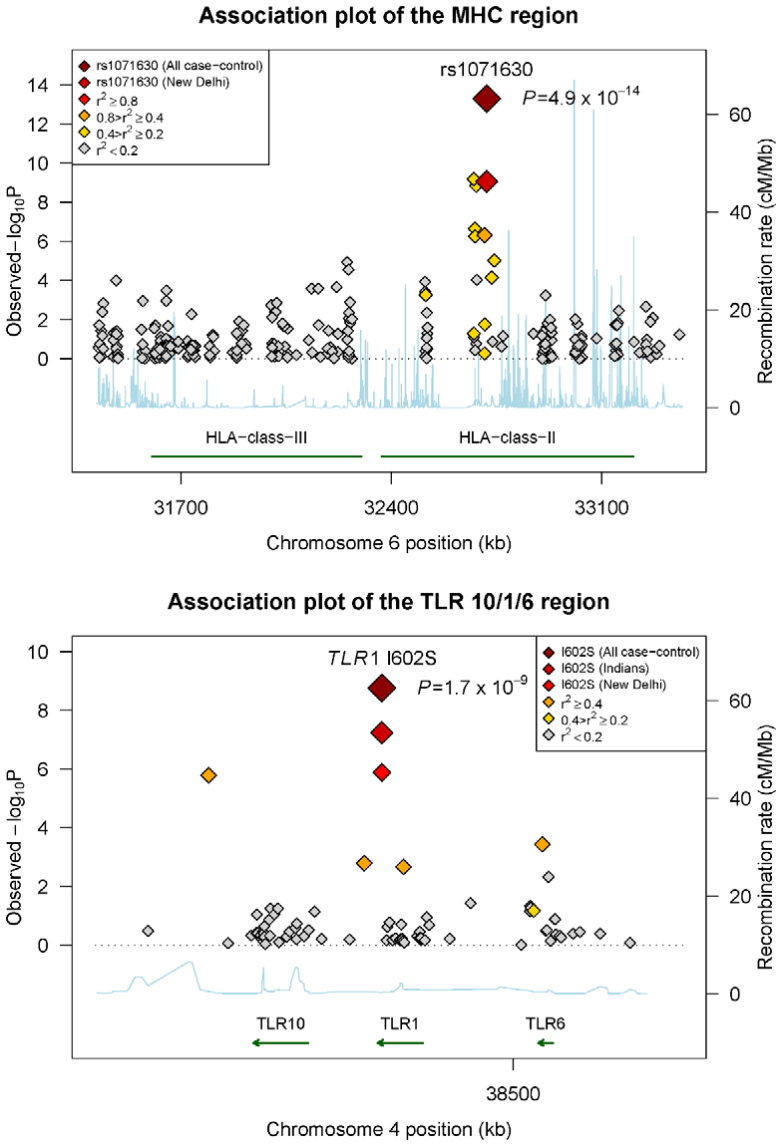
Association plots of the *HLA class II* and *TLR 10/6/1* regions with leprosy susceptibility. The association statistics are presented as negative logarithms of the *P*-values. The color scales correlates with the linkage disequilibrium *r* ^2^ with the most associated SNP in the respective region.

We genotyped variants with *P*<1×10^−4^ in the primary association analysis, using the Sequenom MassArray primer extension assay in an independent case-control cohort recruited from Kolkata (220 cases and 162 controls), West Bengal as part of the replication study. To further minimise the possibility of population stratification resulting in spurious association, we undertook a family-based study recruited from Kumbakonam (N = 941 with 161 families), Tamil Nadu in south India which is robust to population substructure [6]. These results provide further support for the *HLA* associations, with rs1071630 located in *HLA-DQA1* showing convincing evidence of association across these populations (case-control *P* = 4.9×10^−14^, OR = 0.43, 95% CI = 0.35–0.54, **Table 1** **,** **Figure 2** **, Figure S3 and Table S4**). Strong evidence of association was also observed with rs9270650 in *HLA-DRB1* (case-control *P* = 3.1×10^−11^, OR = 2.24, 95% CI = 1.76–2.86, **Table 1** **, Figure S3 and Table S4**). Despite the more significant associations observed in the recessive models as compared to the dominant models, the allelic tests remained most significant suggesting that the alleles may be acting with a multiplicative effect (**Table S5**). The associations remained highly significant after corrections for age and gender in logistic regression models (**Table S5**). Statistical tests of heterogeneity showed similar effect sizes between the multibacillary and paucibacillary subtypes (**Table S6**). These results are consistent with a recently published study conducted in the Han Chinese population, showing association at the same locus with a similar effect size [7]. Conditional logistic regression analysis suggested that neither of these two variants can alone account for the observed association (**Table S7**), but instead form bi-variate haplotypes highly associated with disease states (haplotype TC, case-control *P* = 7.3×10^−15^, OR = 0.41, 95% CI = 0.33–0.52; haplotype CT, case-control *P* = 5.2×10^−12^, OR = 2.20, 95% CI = 1.75–2.76, **Table S8**). Individuals homozygous for the risk alleles at both loci are more than eight times more susceptible to leprosy than their homozygous protective counterparts (**Figure S4 and Table S9**).

### Toll-like receptor 1 (*TLR1*) region

Consistent direction of effect was also observed at the Toll-like receptor (*TLR*) gene cluster at chromosome 4p14. The association peaks at a non-synonymous variant rs5743618 (*TLR1* I602S/T1805G), which affects receptor translocation to the cell surface [8], with a significant protective association against leprosy in both New Delhi (*P* = 1.3×10^−6^, OR = 0.27, 95% CI = 0.15−0.47) and Kolkata (*P* = 0.012, OR = 0.40, 95% CI = 0.20–0.83, **Table 1** **, Table S5**). The combined statistic from these two case-control populations showed strong evidence of association (*P* = 5.7×10^−8^, OR = 0.31, 95% CI = 0.20–0.48). The Kumbakonam family study showed a consistent direction of effect (*P* = 0.090, OR = 0.61, 95% CI = 0.35–1.09). Analyzed together with a small reported set of 57 cases and 90 controls in Turkey [8], the best estimate of risk for the 602S allele is OR = 0.37 (95% CI = 0.26–0.51) with an overall *P* = 1.7×10^−9^ for the case-control studies (**Table 1** **,** **Figure 2** **and Table S5**). This association is the strongest among all 75 SNPs genotyped in the 80 kb flanking region, and accounts for the observed association in the locus (**Table S10**). The associations remained significant after corrections for age and gender (**Table S5**), with similar effect sizes between the multibacillary and paucibacillary subtypes (**Table S6**). Haplotypic analysis identified a haplotype containing the 602S variant that associated significantly with leprosy susceptibility (CTTTCT, *P* = 1.3×10^−6^, OR = 0.29, 95% CI = 0.18–0.48, **Figures S5** and **S6**, **Table S11**), although this magnitude of effect is similar to the single locus association suggesting this effect is mainly driven by the 602S association. In fact, this *TLR1* variant shows the most consistent association after *HLA* among the 2,092 genes tested in our microarray. However, this association was not observed in the recent GWA study [7], likely due to the absence of this SNP on the array utilized by the Zhang *et al*, and the low power to detect the association by proxy SNPs due to the low allele frequency (1.7%) in the Han Chinese population.

### Previous candidates in leprosy susceptibility

The gene-centric microarray allows genic SNPs to be interrogated at a high density including putative leprosy susceptibility candidates (**Table 2**). Notably, evidence of association was observed at SNPs flanking *MICA* (rs12660741, *P* = 9.9×10^−5^) and *TNF* (rs3093662, *P* = 3.2×10^−4^), both located on chromosome 6. These associations remained nominally significant after correcting for the *HLA* variants rs9270650 and rs1071630. These data suggest that these genes may be involved in immunity against *M. leprae*. However, we did not replicate the reported association with the *LTA* promoter variant [9] (rs2239704/*LTA* +80, *P* = 0.20), or with the regulatory variants in *PARK2/PACRG* within the New Delhi case-control study as previously described [10]. Despite consistent replication at the Chromosome 13 locus (*C13orf31* and *CCDC122*), we did not observe any significant association with variants in *NOD2*, *TNFSF15* or *RIPK2* [11] that were identified in the recent GWA study [7]. The differences may be due to population specific effects which have been described in other diseases [12] or differences in allele frequencies.

**Table 2:** Association statistics of SNPs in genes previously reported to associate with susceptibility to leprosy.

Chr	Gene	No. of SNPs	Best SNP	OR	95% CI	*P*-value	Bonferroni *P-*value
1	*IL10*	13	rs1554286	1.52	1.15–1.99	0.003	0.039
2	*SLC11A1*	9	rs13062	1.39	1.03–1.88	0.032	0.288
4	*TLR2*	8	rs1339	0.72	0.47–1.09	0.116	0.928
5	*IL12B*	15	rs919766	2.82	1.22–6.52	0.011	0.165
6	*MICA*	13	rs12660741	2.15	1.45–3.18	9.9×10^−5^	0.001
6	*MICB*	8	rs3828917	0.32	0.16–0.66	0.001	0.008
6	*LTA*	15	rs1121800	1.20	0.92–1.56	0.19	1.000
6	*TNF*	15	rs3093662	0.29	0.13–0.63	3.2×10^−4^	0.005
6	*PARK2* [10]	2	rs6455767	0.73	0.46–1.15	0.177	0.354
6	*PACRG* [10]	1	rs9458710	1.27	0.68–2.36	0.454	0.454
9	*TLR4*	28	rs7044464	1.57	1.05–2.35	0.029	0.812
12	*VDR*	62	rs2254210	0.60	0.41–0.88	0.008	0.496
16	*NOD2* [27]	27	rs4785224	0.65	0.46–0.92	0.012	0.324
19	*IL12RB1*	9	rs436857	0.77	0.51–1.16	0.207	1.000

This table shows the number of SNPs and the best SNP in each gene, with the Bonferroni *P*-value corrected with the number of SNPs in the region. Results previously presented are referenced as denoted.

### Population differentiation at *TLR1*

**Figure 3:**
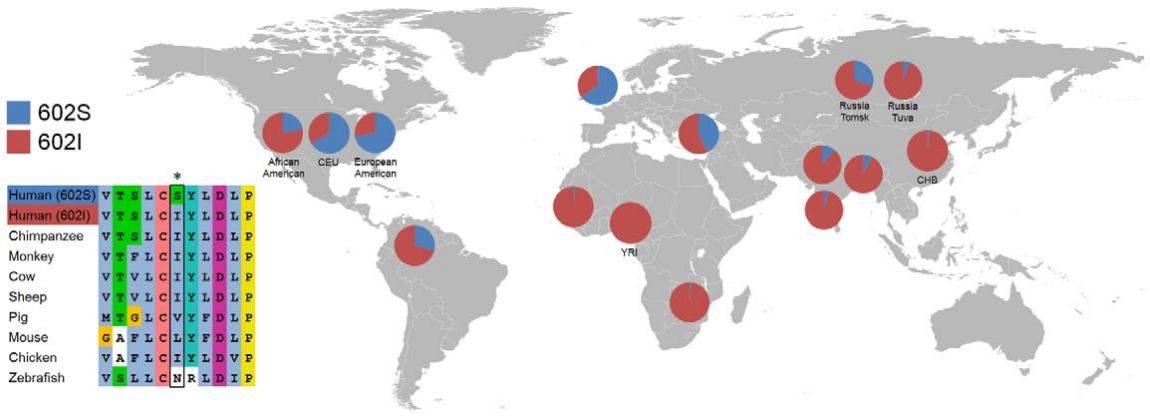
Population differentiation at *TLR1* I602S. The pie charts show the global distribution of *TLR1* I602S allele frequency. Side panel shows the multiple amino acid sequence alignment around the polymorphism in different species.

Infectious diseases have been a major selective force during human evolution [13], [14] and have contributed to the diversity of the mammalian *HLA* genes [15]. Several studies have shown that this *TLR* gene cluster is highly differentiated between populations [16], [17], [18]. To further investigate the geographic distribution of this locus, we genotyped 12 SNPs in this region in 1,463 individuals from 6 populations (New Delhi, Kolkata and Kumbakonam from India, Malawi, Gambia and United Kingdom, **Table S1**). We observed a high degree of population differentiation at this locus [16] (**Figure S7**), which peaks at the I602S variant with a F_ST_ value higher than any other variants in this locus (**Table S12**). To compare the degree of differentiation with the rest of the genome, we genotyped I602S in the CEU, CHB and YRI populations [19] and observed extreme differentiation (F_ST_ = 0.55) among over 3 million polymorphic SNPs in the genome (>99th percentile). Mapping the global frequency of the I602S mutation in 15 different populations reveals that the protective serine allele is derived and rare in Africa, but expands in frequency to become the dominant allele in Europe (**Figure 3** **and Figure S8**). Today, more than 40% of individuals of European descent are homozygous for this mutation. The ancestral isoleucine allele is crucial for protein translocation to the cell membrane, which is abrogated by the hydrophilic serine substitution resulting in a hypo-functional phenotype [8], [20], [21] that associates with protection against an important pathogen. Together with a recent study showing signature of selection at this locus [22], these suggest that mycobacteria may have contributed to the extreme population differentiation at this locus. This provides insight into the host-pathogen relationship between human and *M. leprae*.

## Discussion

Despite the long history of leprosy and medical advances, leprosy remains a scourge in many parts of the world. Our understanding of the pathogenesis of *M. leprae* and its interaction with humans is limited, in part due to the inability to culture the bacterium *in vitro*. For this reason, there is greater reliance on genetics to understand the disease and its causative pathogen. Some progress has been made to identify genetic loci which associate with susceptibility to leprosy; however the numbers of candidates showing consistent replication across populations are few. We here conduct a population based case-control association study, surveying genetic variants in more than 2,000 genes throughout the genome.

Our gene-centric study identified genetic variants at *TLR1* and *HLA-DRB1/DQA1* as major determinants of leprosy susceptibility. These associations showed a consistent direction of effect across the populations under study, including the family samples from Kumbakonam in South India although the two-sided association statistic was borderline for the *TLR1* variant (**Table 1**). These results minimize the chance that the associations are spurious, because the transmission disequilibrium test is known to be robust to population stratification.

The human TLR1 has been previously shown to mediate immune response to *M. leprae* [21]. Our results advance our understanding of human immunity to *M. leprae*. Firstly, this present study is the first to show the consistent effect of *TLR1* on leprosy susceptibility, at a significance level exceeding a genome-wide threshold. Secondly, the *TLR1* association is the most significant after the *HLA* class II locus, of the greater than 2,000 loci surveyed. Although this result does not exclude the possibility of other genes involved in leprosy, it does support that the innate immune system, activated by the TLR receptors, plays a significant role in the defence against *M. leprae* in humans. Thirdly, the hydrophilic serine substation (*TLR1* I602S) has been shown to result in a functional knockout phenotype [8], [23]. Although this mutation is rare in Africa and Asia, our data suggest that a significant proportion of individuals of European descent are homozygous for this knockout variant. These functional TLR1 knockout individuals have a normal immunological phenotype and are protected against leprosy, suggesting that *M. leprae* may have utilized TLR1 as part of its pathogenesis mechanisms, as certain bacteria are known to produce homologues of the TLR1 signaling domain to subvert the innate immune responses [24]. Collectively, these results suggest that modulation of the TLR1 pathway may be valuable in future treatment of mycobacterial diseases without significant side effects.

When we compare these results to the recently published GWA study [7], we observed both consistent and discrepant results with the different genetic loci. Reassuringly, both studies confirm the class II *HLA* locus as a major leprosy susceptibility locus, a finding consistent with early studies reporting association of *HLA* with leprosy [25], [26]. Consistent findings were also observed for genetic variants at *C13orf31* and *CCDC122* [27]. However, separate genotyping of the reported SNPs did not replicate the associations at the *NOD2*, *TNFSF15* or *RIPK2* genes [27]; nor did the Zhang *et al*. study report any notable association at the *TLR1* locus [7]. The disparity at the *TLR1* locus is likely due to the low allele frequency of *TLR1* I602S, and that the haplotype carrying this variant would have low power to detect the association. For the other associated genes (*NOD2*, *TNFSF15* and *RIPK2*) there could be a number of reasons for the non-replication. The reported effect sizes of variants in these genes are moderate compared to the *HLA* and chromosome 13 locus. Assuming these effect estimates are real, the present study will have less power to detect these associations. Another possibility is a population specific effect whereby variants in these genes confer susceptibility in the Chinese population but not in the Indians. Given the significant differences in the genetic composition between the Indian and Chinese [28], this may have contributed to the heterogeneous signals between the studies. Previous studies have described population specific genetic effects for other diseases [12]. Furthermore, despite the relative homogenous population of *M. leprae*, the 16 mycobacterial subtypes highly correlated with the geography can be readily identified [29]. Given the highly specialized nature of the immune system, these mycobacterial strains may activate the host immune response in slightly different ways, accounting for overlapping yet some discordant results between the studies. Finally, we cannot exclude the possibility that *NOD2*, *TNFSF15* and *RIPK2* are not true leprosy susceptibility loci.

This study provides insight into the host-pathogen relationship between human and *M. leprae*. Our data showed that the *TLR1* locus is more highly differentiated as compared with the rest of the genome. Although the historical selective agents behind this differentiation are difficult to ascertain, our data suggests that mycobacterial infections may contribute to such a high degree of differentiation. Several studies have suggested that leprosy may be associated with reduced reproductive fitness [30], [31], and the all-cause mortality of lepromatous leprosy was reported to be at least 3.5 times of the general populations [32], [33]. These factors, together with the long human co-existence with *M. leprae* [34] and intense social stigma associated with the disease [35], suggest that leprosy may have contributed to the present global distribution of human *TLR1*. Other infectious diseases especially tuberculosis could have also contributed, given the overlapping antigenic repertoire and immunogenicity of mycobacterial species, despite a lack of association in a Gambian case-control population due to low allele frequency (**Table S13**). However, given the lack of data on concurrent selective events, this hypothesis remains difficult to prove and other mechanisms are possible including genetic drift, population migration or bottlenecks, or a combined effect of two or more of these mechanisms.

In conclusion, this study suggests that *TLR1* and *HLA-DRB1/DQA1* are important genetic determinants of susceptibility to leprosy. This study also shows a protective effect against an important pathogen with a hypo-functional TLR1 variant. These observations further highlight the key role of the TLRs in infectious diseases [36], [37] and suggest that modulation of the TLR1 pathway may be valuable in future treatment of mycobacterial diseases.

## Materials and Methods

### Ethics statement

For the New Delhi cohort of samples, informed written consents following the Indian Council of Medical Research (ICMR) specification were obtained from all the individuals whose blood samples were collected. The study was approved by the Jawaharlal Nehru University ethics committee. For the Kolkata cohort of samples, approval was obtained from the ICMR and consent was obtained from the Tropical School of Calcutta and the Swasti Blood Donor Centre at Kolkata. For the Kumbakonam samples, ethical approval was obtained from the ICMR, New Delhi and Hindu Mission Hospital in Kumbakonam.

### Subjects

We genotyped 258 leprosy patients and 300 controls from New Delhi [38]. After quality control filtering with call rates (>95% for SNPs and >90% for samples), minor allele frequency (>1%), Hardy-Weinberg equilibrium (*P*>1×10^−6^), relatedness (<20%), heterozygosity (22–27%) and outlying ancestry (within 97.5^th^ percentile), the genotype rate was 99.5% with separate multi-dimensional scaling (MDS) and principal component analysis (PCA) showing minimal population substructure in the 448 individuals (209 cases and 239 controls) carried forward for analysis. The replication studies included 220 leprosy patients and 162 controls from Kolkata, and 941 individuals from 246 families from Kumbakonam [39], with which 299 individuals (168 cases and 131 controls) from Kolkata and 852 individuals from Kumbakonam were included after quality control filtering. Diagnosis and classification of leprosy was based on clinical and microscopic examination of the skin lesions. Patients were classified either as multibacillary (MB, with bacterial index >0), a group that included patients with lepromatous (LL), borderline lepromatous (BL) and borderline (BB) leprosy, or as paucibacillary (PB, with bacterial index of 0) which included patients with borderline tuberculoid (BT) and tuberculoid (TT) leprosy. Paucibacillary leprosy was characterized by the presence of large well-defined skin lesions which were less than five in number, dry and with almost 90–100% loss of sensation. Multibacillary leprosy was characterized by the presence of six or more skin lesions that were smaller in size, tending to be bilaterally symmetrical with about 10–40% loss of sensation. The study was approved by the relevant ethics committee. Informed consent was obtained from individuals whose blood samples were collected. In addition, we have also genotyped a total of 1,463 individuals from the Kumbakonam, India, Gambia, Malawi, United Kingdom, Tomsk and Tuva of Russia, and HapMap CEU, CHB and YRI populations [19] for the population differentiation study.

### Genotyping and quality control

All individuals in the New Delhi cohort were genotyped with the Illumina IBC gene-centric 50 K array [5]. The data quality control and analysis were performed using PLINK [40]. Multi-dimensional scaling (MDS) and principal component analysis (PCA) were carried out with PLINK and EIGENSTRAT to remove population outliers. The microarray genotypes more than 48,000 SNPs distributed in approximately 2,100 genes throughout the genome, including 3,470 non-synonymous markers. A total of 209 leprosy cases and 239 controls were carried forwarded for analysis. The SNPs in the replication studies and the population differentiation analysis were genotyped using the Sequenom MassArray primer extension assay. The *TLR1* I602S polymorphism was additionally genotyped with a restriction enzyme digestion assay (*PstI*), and the rs1071630 with a primer induced restriction assay (*MlyI*).

### Analysis

Separate MDS and PCA analyses were performed in PLINK to visualize the population structure in the New Delhi cohort (**Figure S1**), and individuals with outlying ancestry were removed. The analysis was performed in the resultant set of individuals. The primary test of association in the New Delhi and Kolkata cohorts was carried out with the Pearson's χ2 allelic test, Cochran-Armitage trend test and logistic regression (**Table S5**); although the allelic test statistics were used in the main text and tables unless otherwise specified. The transmission-disequilibrium-test was used for the Kumbakonam family cohort. Both analyses were performed in PLINK. The primary association with the allelic test was performed without any correction. However, the associations at *TLR1* and *HLA-DRB1/DQA1* were further tested in a logistic regression model with correction for age, gender and the first MDS component (**Table S5**). As this study involves genotypes of genic instead of random SNPs, the quantile-quantile (QQ) plot was generated with comparison to the statistics of the same SNPs in the WTCCC Crohn's disease, rheumatoid arthritis and type 1 diabetes cohorts in addition to the expected values under the null hypothesis (**Figure S2**), which suggests that the statistics are not inflated by population substructure. The Mantel Haenszel statistics was used to combine data from case-control cohorts, but only when there was no significant heterogeneity in odds ratio between studies indicated by the Woolf's test. The haplotypic analysis was performed by Haploview, with the haplotypes reconstructed according to the confidence interval definition [41]. The fixation index F_ST_ was used for the population differentiation analysis. Let *pi* denote the allele frequency of the ith population and *p-bar* denote the mean allele frequency, the F_ST_ value is given by
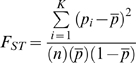
where *n* is the number of populations in comparison. The haplotype diversity analysis was performed with Haplosuite [42].

### Accession numbers

#### Genes

Toll-like receptor 1; *TLR1* (NCBI GeneID:7096); HLA-DRB1 major histocompatibility complex, class II, DR beta 1; *HLA-DRB1* (NCBI GeneID:3123); HLA-DQA1 major histocompatibility complex, class II, DQ alpha 1; *HLA-DQA1* (NCBI GeneID:3117); interleukin 10; *IL10* (NCBI GeneID:3586); solute carrier family 11; *SLC11A1* (NCBI GeneID:6556); Toll-like receptor 2; *TLR2* (NCBI GeneID:7097); interleukin 12b; *IL12B* (NCBI GeneID:3593); MHC class I polypeptide-related sequence A; *MICA* (NCBI GeneID:4276); MHC class I polypeptide-related sequence B; *MICB* (NCBI GeneID:4277); lymphotoxin alpha; *LTA* (NCBI GeneID:4049); tumor necrosis factor; *TNF* (NCBI GeneID:7124); Parkinson disease 2, parkin; *PARK2* (NCBI GeneID:5071); PARK2 co-regulated; *PACRG* (NCBI GeneID:135138); Toll-like receptor 4; TLR4 (NCBI GeneID: 7099); vitamin D receptor; *VDR* (NCBI GeneID:7421); interleukin 12 receptor, beta 1; *IL12RB1* (NCBI GeneID:3594); nucleotide-binding oligomerization domain containing 2; *NOD2* (NCBI GeneID:64127); receptor-interacting serine-threonine kinase 2; *RIPK2* (NCBI GeneID: 8767); tumor necrosis factor (ligand) superfamily, member 15; *TNFSF15* (NCBI GeneID:9966); chromosome 13 open reading frame 31; *C13orf31* (NCBI GeneID: 144811); coiled-coil domain containing 122; *CCDC122* (NCBI GeneID: 160857).

## Supporting Information

Figure S1 Multi-dimensional scaling (MDS, left) and principal component analysis (PCA, right) of New Delhi Indian and HapMap samples. The first two components from the pairwise matrix were plotted for the New Delhi individuals (black) in this study, along with 210 unrelated samples from the International HapMap Project (CEU - red, CHB+JPT - blue, YRI - green) [19]. Crosses in grey represented samples removed from the analysis due to outlying ancestry.
(0.08 MB JPG)

Figure S2 Quantile-quantile (QQ) plot of overlapping SNPs between the New Delhi leprosy and WTCCC studies. The dotted line shows expected association statistics under the null hypothesis. The coloured triangles represent the association statistics of the intra-genic SNPs in the New Delhi leprosy and the WTCCC Crohn's disease, rheumatoid arthritis and type 1 diabetes cohorts respectively. The observed statistics for the WTCCC Crohn's disease cohort using random SNPs are also shown in dark green.
(1.44 MB PDF)

Figure S3 Forest plots showing the association statistics in the primary and replication studies. The statistics were calculated with the Pearson's χ^2^ allelic test with 1 degree of freedom.
(0.48 MB JPG)

Figure S4 The odds of leprosy infection against the double homozygous protective group. The graph shows the odds of infection among individuals carrying zero to four risk alleles for the SNPs rs9270650 and rs1071630.
(0.00 MB PDF)

Figure S5 The pattern of linkage disequilibrium at the *TLR 10/6/1* region, with the shades of red and black indicating the corresponding D' and *r* ^2^ values respectively. The SNP *TLR1* I602S (rs5743618) is labelled in green.
(0.86 MB JPG)

Figure S6 The pattern of linkage disequilibrium around the SNP *TLR1* I602S (rs5743618). The haplotype was reconstructed using the confidence interval definition in Haploview [41].
(0.11 MB JPG)

Figure S7 Visualization of the haplotype diversity in the *TLR 10/1/6* region. The figure represents the haplotype distribution across the six populations, where each chromosome is either mapped uniquely to one of twelve possible canonical haplotypes or mapped as a mosaic of the twelve haplotypes. The canonical haplotypes are defined as the haplotypic forms that most of the 2,392 chromosomes are similar to. Each color represents a unique canonical haplotype and the same color scheme is used for the six panels. The purple colour represents the canonical haplotype containing the *TLR1* I602S variant.
(0.30 MB JPG)

Figure S8 Pairwise F_ST_ of *TLR1* I602S in 15 different populations. Apart from the six population described, we additionally genotype three HapMap populations (CEU, CHB and YRI) [19] and two Russian populations (Tomsk and Tuva) and retrieved data from more populations from literature [8], [43]. The yellow-to-red scale indicates low-to-high F_ST_ value and bars indicate frequency of the 602S allele.
(0.44 MB JPG)

Table S1 Study design. Outline of numbers of individuals in each of the analyses performed in this study. Retrieved data from published literature or public datasets are referenced as indicated
(0.05 MB DOC)

Table S2 Quality control criteria for the 50 K microarray genotyping for both samples and SNPs.
(0.03 MB DOC)

Table S3 Association statistics of all SNPs with *P*<1×10^−4^ in the Pearson's χ^2^ test in the primary association analysis in the New Delhi cohort. Statistically significant associations in the New Delhi cohort were verified with a logistic regression model, correcting for sex and age as covariates.
(0.09 MB DOC)

Table S4 Association statistics of SNPs in the replication cohorts with susceptibility to leprosy. All SNPs with nominal *P*<0.05 with the same direction in either of the replication cohorts were highlighted in bold.
(0.10 MB DOC)

Table S5 Full genotypic counts and association statistics for SNPs rs1071630, rs9270650 and rs5743618 (I602S) under different genetic models. All the models in Pearson's χ^2^ represent 1-df test except the genotypic model which has 2 degrees of freedom.
(0.06 MB DOC)

Table S6 Stratified association analyses (leprosy type and age) and linear regression analysis (age of onset) for the three replicated SNPs rs1071630, rs9270650 and rs5743618 (I602S) at *HLA-DRB1/DQA1* and *TLR1*. The Pearson's χ^2^ test was used for the stratified analyses, and the test of heterogeneity was performed with Woolf's test.
(0.05 MB DOC)

Table S7 Conditional logistic regression analysis of SNPs associated with leprosy susceptibility (*P*<0.05) at the *HLA-DRB1/DQA1* locus.
(0.04 MB DOC)

Table S8 Bi-variate haplotypic analysis with SNPs rs9270650-rs1071630 in the *HLA*. The *P*-value statistics in each population were given by the likelihood ratio test in PLINK [40]. The combined values were given by the Mantel Haenszel statistics after confirming non-heterogeneity with Woolf's test of heterogeneity.
(0.07 MB DOC)

Table S9 Allelic dosage analysis for number of risk alleles at SNPs rs9270650 and rs1071630 on susceptibility to leprosy.
(0.03 MB DOC)

Table S10 Conditional logistic regression analysis of SNPs associated with leprosy susceptibility (*P*<0.05) on the *TLR1* I602S polymorphism.
(0.03 MB DOC)

Table S11 Haplotypic analysis of the *TLR 10/1/6* locus using SNPs rs7660429, rs11725309, rs10004195, rs7663239, rs5743618 (I602S) and rs4833095 in the New Delhi leprosy cohort.
(0.03 MB DOC)

Table S12 Pairwise and global F_ST_ statistics of the 12 SNPs genotyped in the six populations recruited from New Delhi, Kolkata, Kumbakonam of India, Malawi, The Gambia and the United Kingdom. The SNP rs5743618 (*TLR1* I602S) is highlighted in bold.
(0.05 MB DOC)

Table S13 Association statistics of rs5743618 (*TLR1* I602S) in a Gambian tuberculosis case control population.
(0.03 MB DOC)

## References

[1] Chakravartti M, Vogel F (1973). A twin study on leprosy..

[2] Shields ED, Russell DA, Pericak-Vance MA (1987). Genetic epidemiology of the susceptibility to leprosy.. J Clin Invest.

[3] Abel L, Vu DL, Oberti J, Nguyen VT, Van VC (1995). Complex segregation analysis of leprosy in southern Vietnam.. Genet Epidemiol.

[4] Bakker MI, May L, Hatta M, Kwenang A, Klatser PR (2005). Genetic, household and spatial clustering of leprosy on an island in Indonesia: a population-based study.. BMC Med Genet.

[5] Keating BJ, Tischfield S, Murray SS, Bhangale T, Price TS (2008). Concept, design and implementation of a cardiovascular gene-centric 50 k SNP array for large-scale genomic association studies.. PLoS ONE.

[6] Cardon LR, Palmer LJ (2003). Population stratification and spurious allelic association.. Lancet.

[7] Zhang FR, Huang W, Chen SM, Sun LD, Liu H (2009). Genomewide association study of leprosy.. N Engl J Med.

[8] Johnson CM, Lyle EA, Omueti KO, Stepensky VA, Yegin O (2007). Cutting edge: A common polymorphism impairs cell surface trafficking and functional responses of TLR1 but protects against leprosy.. J Immunol.

[9] Alcais A, Alter A, Antoni G, Orlova M, Nguyen VT (2007). Stepwise replication identifies a low-producing lymphotoxin-alpha allele as a major risk factor for early-onset leprosy.. Nat Genet.

[10] Malhotra D, Darvishi K, Lohra M, Kumar H, Grover C (2006). Association study of major risk single nucleotide polymorphisms in the common regulatory region of PARK2 and PACRG genes with leprosy in an Indian population.. Eur J Hum Genet.

[11] Wong SH, Hill AV, Vannberg FO (2010). Genomewide association study of leprosy.. N Engl J Med.

[12] Ioannidis JP, Ntzani EE, Trikalinos TA (2004). ‘Racial’ differences in genetic effects for complex diseases.. Nat Genet.

[13] Haldane JBS (1949). Disease and Evolution.. La Ricerca Sci.

[14] Manolio TA, Collins FS, Cox NJ, Goldstein DB, Hindorff LA (2009). Finding the missing heritability of complex diseases.. Nature.

[15] Hill AV, Allsopp CE, Kwiatkowski D, Anstey NM, Twumasi P (1991). Common west African HLA antigens are associated with protection from severe malaria.. Nature.

[16] Pickrell JK, Coop G, Novembre J, Kudaravalli S, Li JZ (2009). Signals of recent positive selection in a worldwide sample of human populations.. Genome Res.

[17] Todd JA, Walker NM, Cooper JD, Smyth DJ, Downes K (2007). Robust associations of four new chromosome regions from genome-wide analyses of type 1 diabetes.. Nat Genet.

[18] The Wellcome Trust Case Control Consortium (2007). Genome-wide association study of 14,000 cases of seven common diseases and 3,000 shared controls.. Nature.

[19] The Wellcome Trust Case Control Consortium (2005). A haplotype map of the human genome.. Nature.

[20] Wurfel MM, Gordon AC, Holden TD, Radella F, Strout J (2008). Toll-like receptor 1 polymorphisms affect innate immune responses and outcomes in sepsis.. Am J Respir Crit Care Med.

[21] Krutzik SR, Ochoa MT, Sieling PA, Uematsu S, Ng YW (2003). Activation and regulation of Toll-like receptors 2 and 1 in human leprosy.. Nat Med.

[22] Grossman SR, Shylakhter I, Karlsson EK, Byrne EH, Morales S (2010). A composite of multiple signals distinguishes causal variants in regions of positive selection.. Science.

[23] Hawn TR, Misch EA, Dunstan SJ, Thwaites GE, Lan NT (2007). A common human TLR1 polymorphism regulates the innate immune response to lipopeptides.. Eur J Immunol.

[24] Cirl C, Wieser A, Yadav M, Duerr S, Schubert S (2008). Subversion of Toll-like receptor signaling by a unique family of bacterial Toll/interleukin-1 receptor domain-containing proteins.. Nat Med.

[25] van Eden W, de Vries RR, Mehra NK, Vaidya MC, D'Amaro J (1980). HLA segregation of tuberculoid leprosy: confirmation of the DR2 marker.. J Infect Dis.

[26] van Eden W, Gonzalez NM, de Vries RR, Convit J, van Rood JJ (1985). HLA-linked control of predisposition to lepromatous leprosy.. J Infect Dis.

[27] Wong SH, Hill AVS, Vannberg FO (2010). Comment on: Genomewide association study of leprosy.. N Engl J Med.

[28] Reich D, Thangaraj K, Patterson N, Price AL, Singh L (2009). Reconstructing Indian population history.. Nature.

[29] Monot M, Honore N, Garnier T, Zidane N, Sherafi D (2009). Comparative genomic and phylogeographic analysis of Mycobacterium leprae.. Nat Genet.

[30] Saporta L, Yuksel A (1994). Androgenic status in patients with lepromatous leprosy.. Br J Urol.

[31] Smith DG, Guinto RS (1978). Leprosy and fertility.. Hum Biol.

[32] Guinto RS, Doull JA, De Guia L (1954). Mortality of persons with leprosy prior to sulfone therapy, Cordova and Talisay, Cebu, Philippines.. Int J Lepr.

[33] Noordeen SK (1972). Mortality in leprosy.. Indian J Med Res.

[34] Monot M, Honore N, Garnier T, Araoz R, Coppee JY (2005). On the origin of leprosy.. Science.

[35] Boldsen JL (2005). Leprosy and mortality in the Medieval Danish village of Tirup.. Am J Phys Anthropol.

[36] Khor CC, Chapman SJ, Vannberg FO, Dunne A, Murphy C (2007). A Mal functional variant is associated with protection against invasive pneumococcal disease, bacteremia, malaria and tuberculosis.. Nat Genet.

[37] Medzhitov R, Janeway C (2000). Innate immunity.. N Engl J Med.

[38] Malhotra D, Darvishi K, Sood S, Sharma S, Grover C (2005). IL-10 promoter single nucleotide polymorphisms are significantly associated with resistance to leprosy.. Hum Genet.

[39] Siddiqui MR, Meisner S, Tosh K, Balakrishnan K, Ghei S (2001). A major susceptibility locus for leprosy in India maps to chromosome 10p13.. Nat Genet.

[40] Purcell S, Neale B, Todd-Brown K, Thomas L, Ferreira MA (2007). PLINK: a tool set for whole-genome association and population-based linkage analyses.. Am J Hum Genet.

[41] Barrett JC, Fry B, Maller J, Daly MJ (2005). Haploview: analysis and visualization of LD and haplotype maps.. Bioinformatics.

[42] Teo YY, Small KS (2010). A novel method for haplotype clustering and visualization.. Genet Epidemiol.

[43] Ma X, Liu Y, Gowen BB, Graviss EA, Clark AG (2007). Full-exon resequencing reveals toll-like receptor variants contribute to human susceptibility to tuberculosis disease.. PLoS ONE.

