# Supplementary figures and images for: Leprosy and the Adaptation of Human Toll-Like Receptor 1
Source: PLoS Pathog. 2010 Jul 1;6(7):e1000979. doi: 10.1371/journal.ppat.1000979 (PMC2895660; doi:10.1371/journal.ppat.1000979)

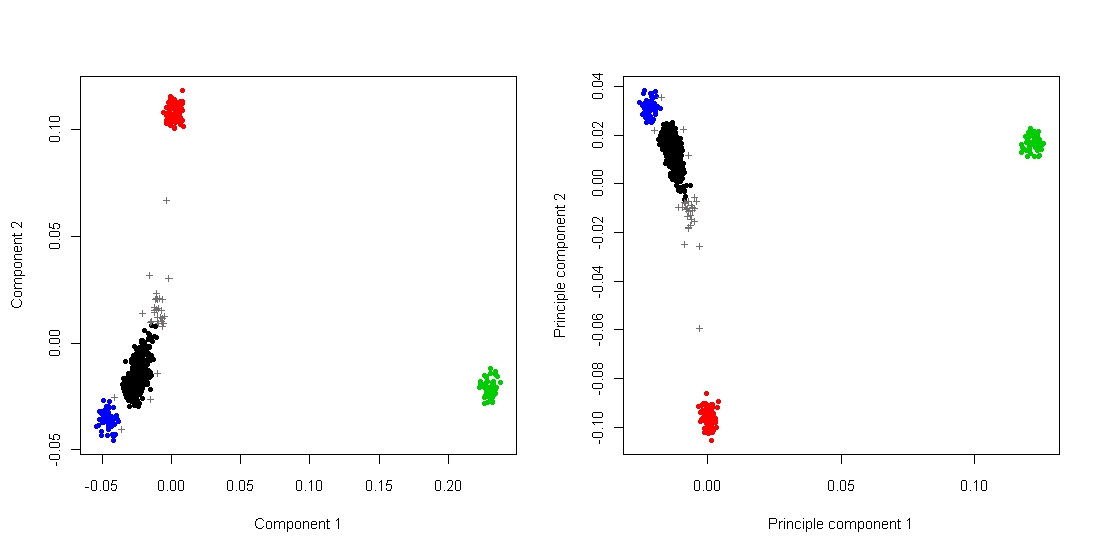

Supplement: Figure S1 — Multi-dimensional scaling (MDS, left) and principal component analysis (PCA, right) of New Delhi Indian and HapMap samples. The first two components from the pairwise matrix were plotted for the New Delhi individuals (black) in this study, along with 210 unrelated samples from the International HapMap Project (CEU - red, CHB+JPT - blue, YRI - green) [19]. Crosses in grey represented samples removed from the analysis due to outlying ancestry. (0.08 MB JPG) [file ppat.1000979.s001.jpg]

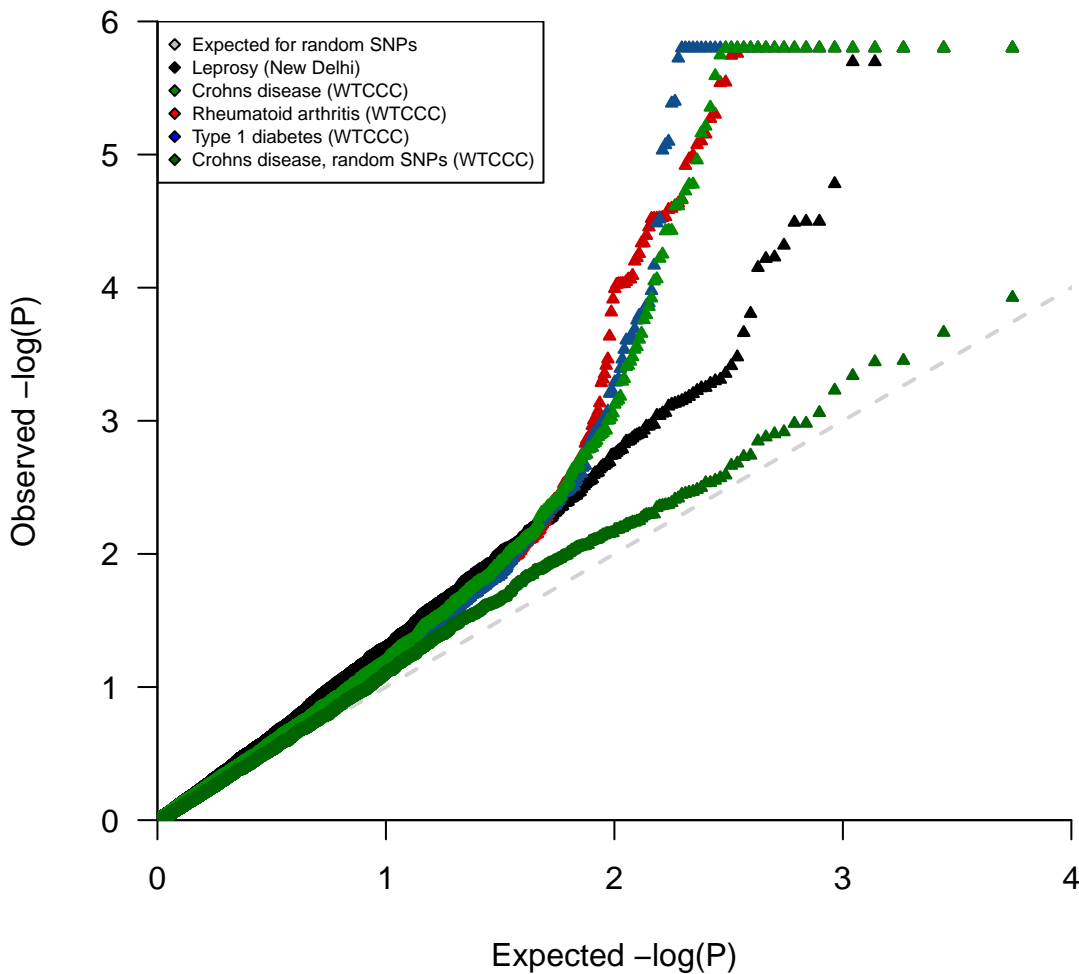

Supplement: Figure S2 — Quantile-quantile (QQ) plot of overlapping SNPs between the New Delhi leprosy and WTCCC studies. The dotted line shows expected association statistics under the null hypothesis. The coloured triangles represent the association statistics of the intra-genic SNPs in the New Delhi leprosy and the WTCCC Crohn's disease, rheumatoid arthritis and type 1 diabetes cohorts respectively. The observed statistics for the WTCCC Crohn's disease cohort using random SNPs are also shown in dark green. (1.44 MB PDF) [file ppat.1000979.s002.pdf]

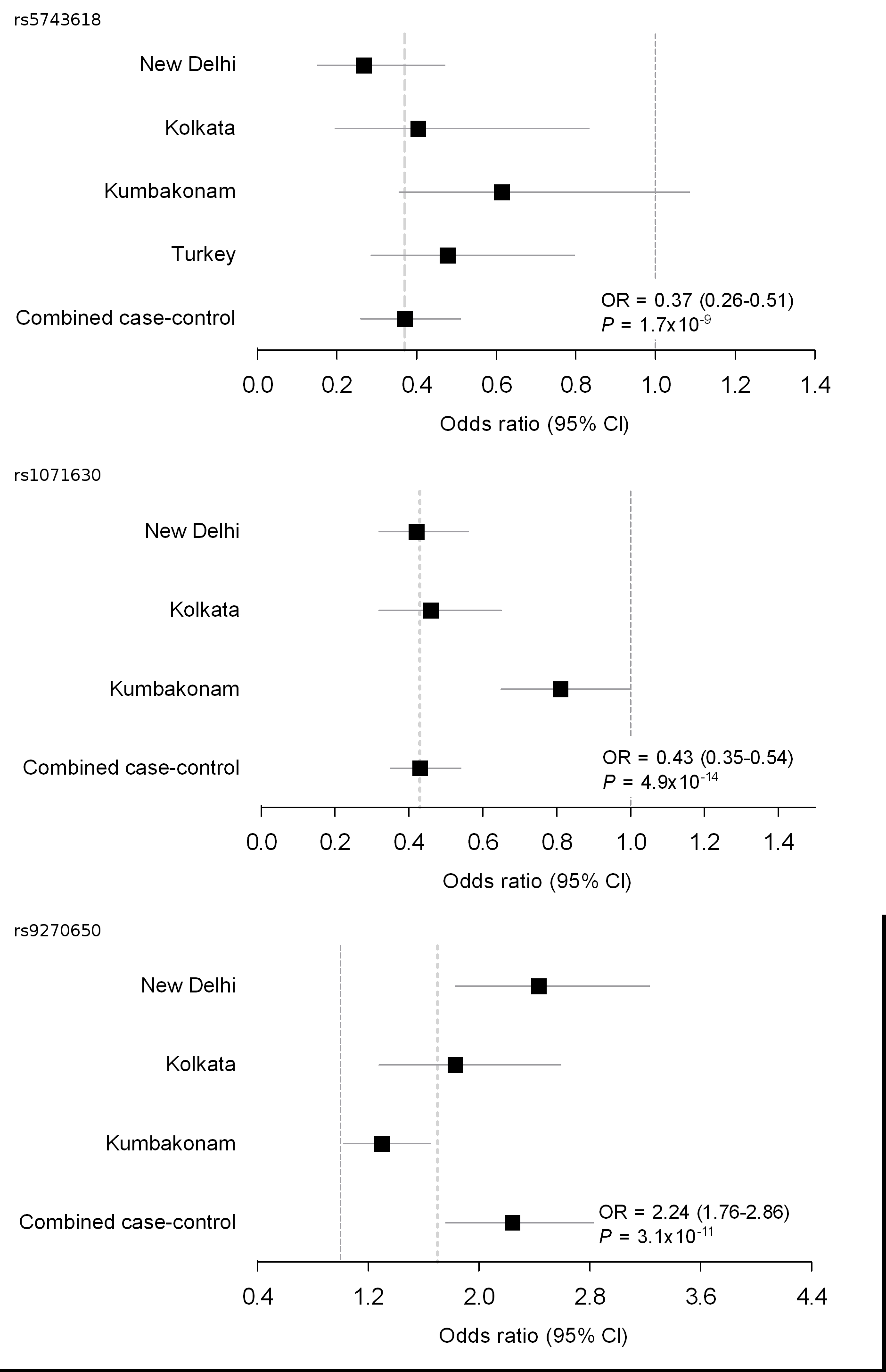

Supplement: Figure S3 — Forest plots showing the association statistics in the primary and replication studies. The statistics were calculated with the Pearson's χ2 allelic test with 1 degree of freedom. (0.48 MB JPG) [file ppat.1000979.s003.jpg]

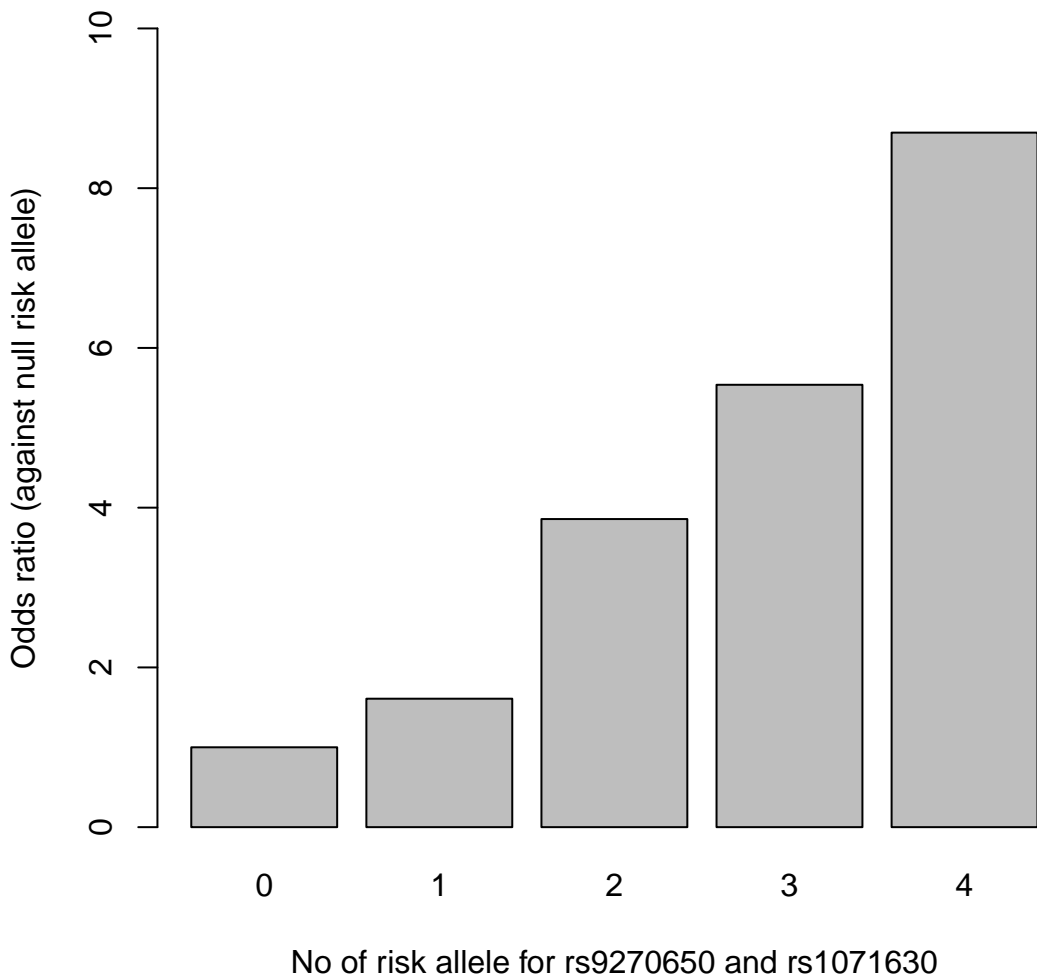

Supplement: Figure S4 — The odds of leprosy infection against the double homozygous protective group. The graph shows the odds of infection among individuals carrying zero to four risk alleles for the SNPs rs9270650 and rs1071630. (0.00 MB PDF) [file ppat.1000979.s004.pdf]

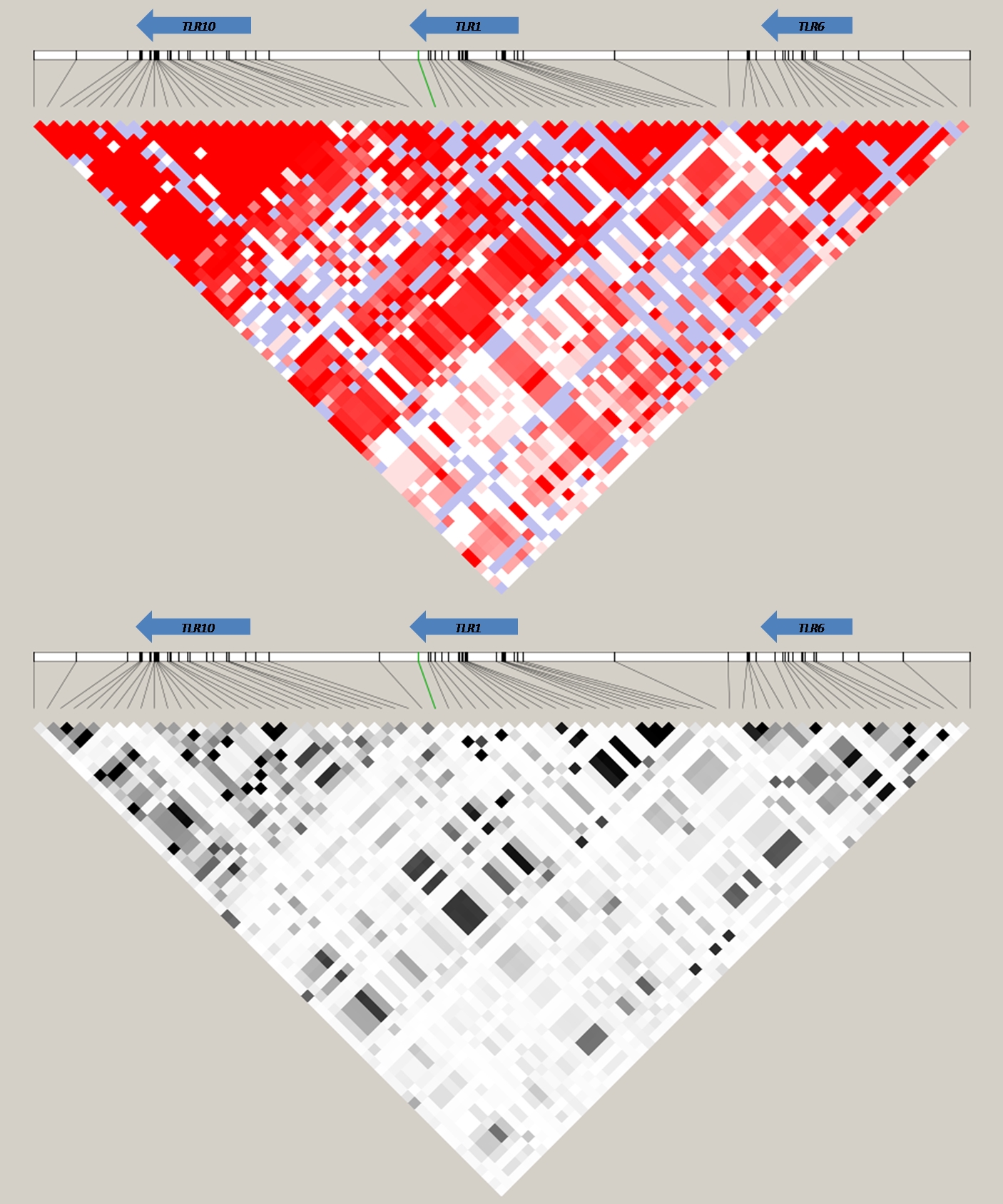

Supplement: Figure S5 — The pattern of linkage disequilibrium at the TLR 10/6/1 region, with the shades of red and black indicating the corresponding D' and r 2 values respectively. The SNP TLR1 I602S (rs5743618) is labelled in green. (0.86 MB JPG) [file ppat.1000979.s005.jpg]

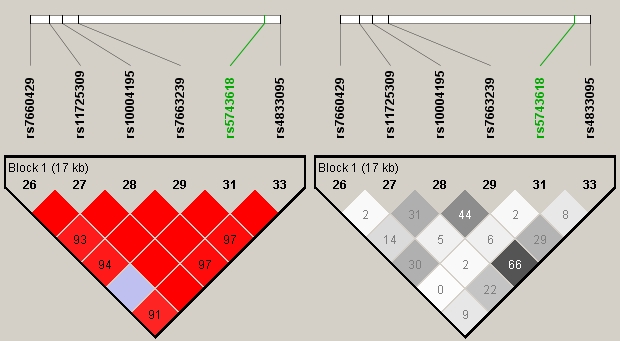

Supplement: Figure S6 — The pattern of linkage disequilibrium around the SNP TLR1 I602S (rs5743618). The haplotype was reconstructed using the confidence interval definition in Haploview [41]. (0.11 MB JPG) [file ppat.1000979.s006.jpg]

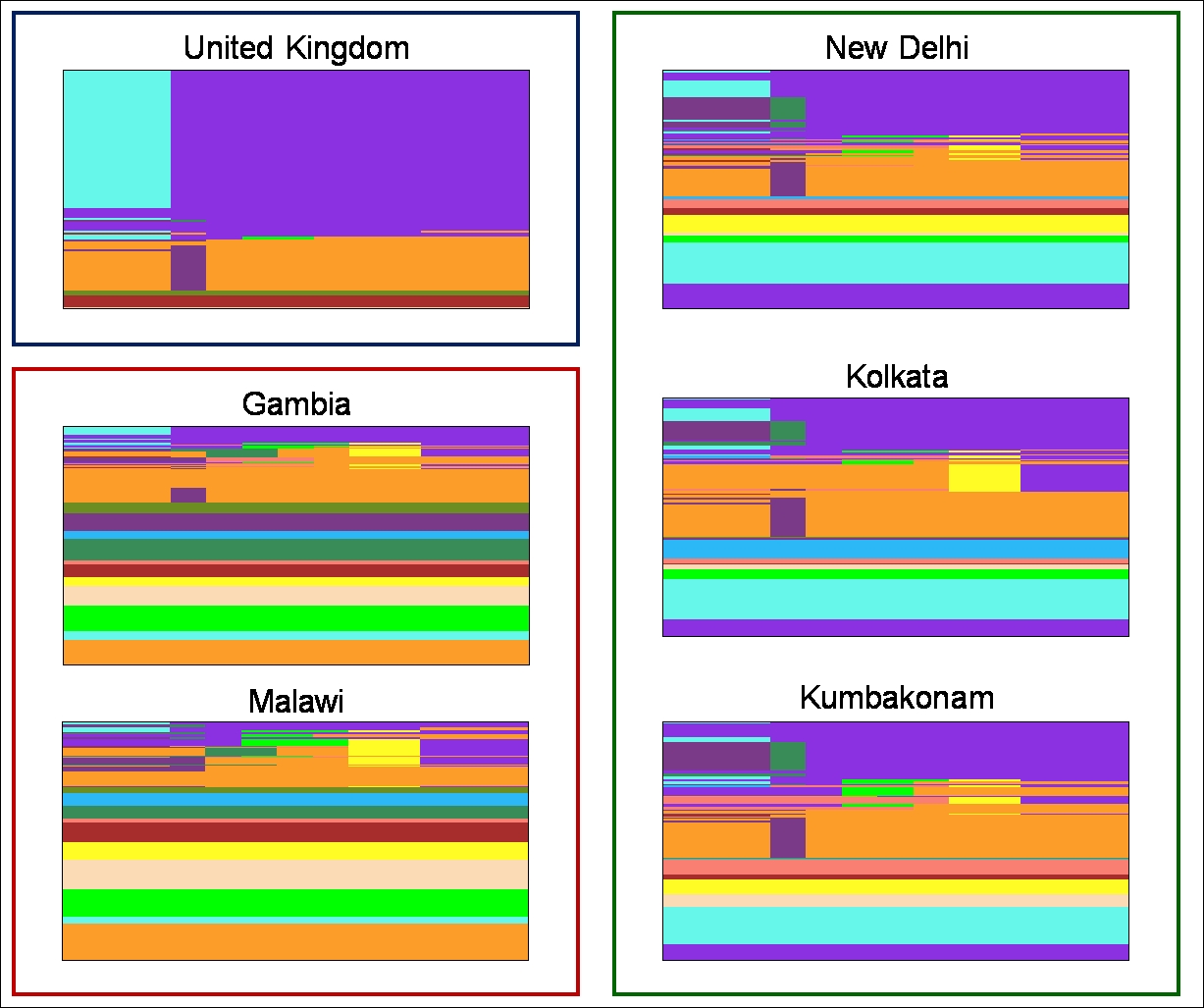

Supplement: Figure S7 — Visualization of the haplotype diversity in the TLR 10/1/6 region. The figure represents the haplotype distribution across the six populations, where each chromosome is either mapped uniquely to one of twelve possible canonical haplotypes or mapped as a mosaic of the twelve haplotypes. The canonical haplotypes are defined as the haplotypic forms that most of the 2,392 chromosomes are similar to. Each color represents a unique canonical haplotype and the same color scheme is used for the six panels. The purple colour represents the canonical haplotype containing the TLR1 I602S variant. (0.30 MB JPG) [file ppat.1000979.s007.jpg]

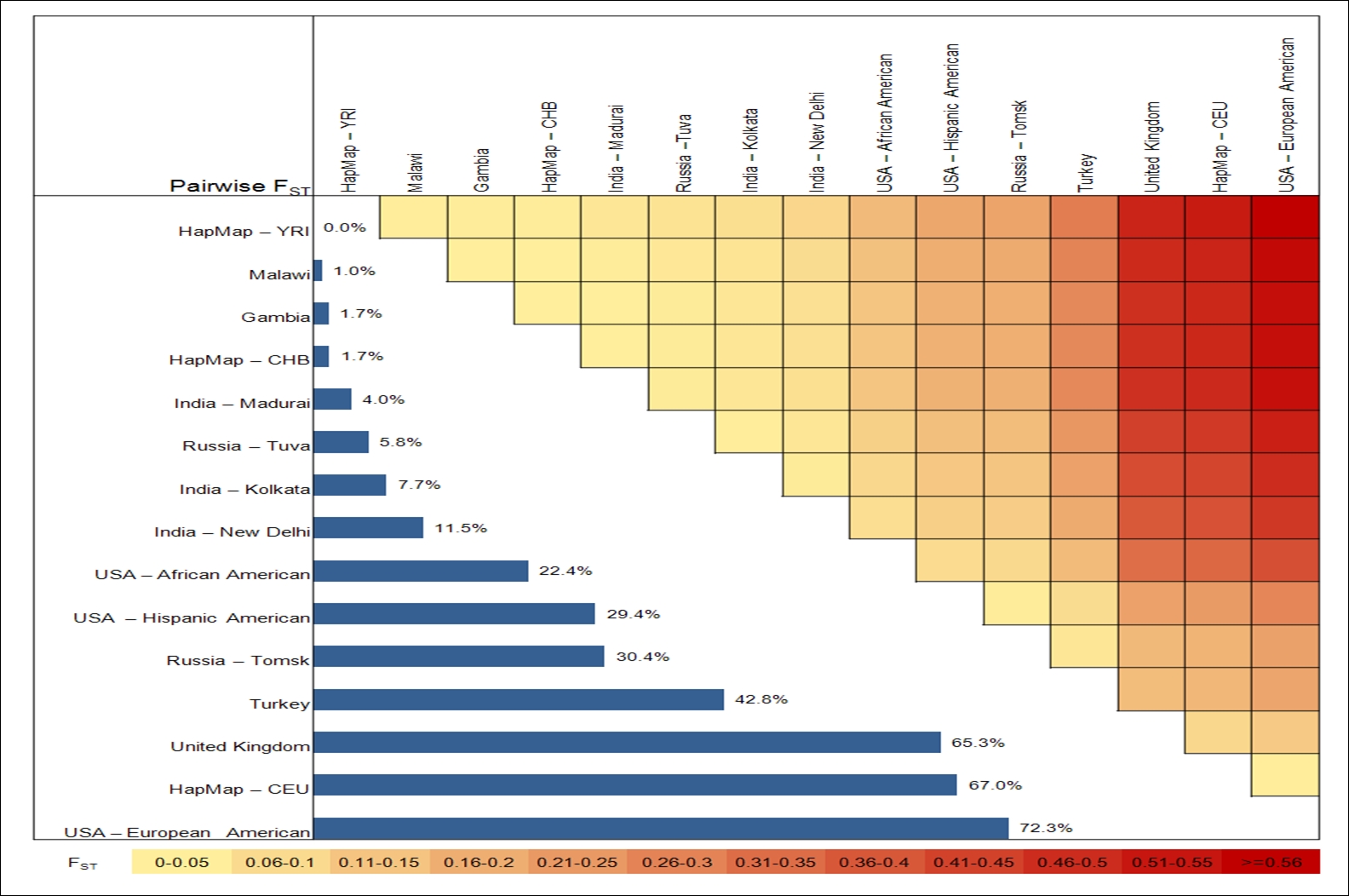

Supplement: Figure S8 — Pairwise FST of TLR1 I602S in 15 different populations. Apart from the six population described, we additionally genotype three HapMap populations (CEU, CHB and YRI) [19] and two Russian populations (Tomsk and Tuva) and retrieved data from more populations from literature [8], [43]. The yellow-to-red scale indicates low-to-high FST value and bars indicate frequency of the 602S allele. (0.44 MB JPG) [file ppat.1000979.s008.jpg]
