# Supplementary material for: Leprosy and the Adaptation of Human Toll-Like Receptor 1
Source: PLoS Pathog. 2010 Jul 1;6(7):e1000979. doi: 10.1371/journal.ppat.1000979 (PMC2895660; doi:10.1371/journal.ppat.1000979)
Supplement: Table S1 — Study design. Outline of numbers of individuals in each of the analyses performed in this study. Retrieved data from published literature or public datasets are referenced as indicated (0.05 MB DOC) [file ppat.1000979.s009.doc]

| **Cohort** | **Location** | **Leprosy** | **Non-leprosy** | **Genotype** |
| --- | --- | --- | --- | --- |
| **Association analysis** | |  |  |  |
| India (1) | New Delhi | 209 | 239 | Present study |
| India (2) | Kolkata | 168 | 131 | Present study |
| India (3) | Kumbakonam | 189 families | | Present study |
| Turkey | Elazig and Antalya | 90 | 57 | Ref [8] |
| **Differentiation analysis** | |  |  |  |
| India (3) | Kumbakonam | - | 251 | Present study |
| Gambia | Banjul | - | 349 | Present study |
| Malawi | Karonga | - | 146 | Present study |
| UK | Oxford | - | 347 | Present study |
| Russia (1) | Tomsk | - | 135 | Present study |
| Russia (2) | Tuva | - | 189 | Present study |
| USA (1) | Houston (Hispanic descent) | - | 114 | Ref [43] |
| USA (2) | Houston (European descent) | - | 110 | Ref [43] |
| USA (3) | Houston (African descent) | - | 194 | Ref [43] |
| HapMap – CEU | Utah (CEU) | - | 111 (max) | Present study, Ref [19] |
| HapMap – CHB | Beijing (CHB) | - | 82 (max) | Present study, Ref [19] |
| HapMap – YRI | Ibadan (YRI) | - | 108 (max) | Present study, Ref [19] |

**Table S1.** Study design. Outline of numbers of individuals in each of the analyses performed in this study. Retrieved data from published literature or public datasets are referenced as indicated.
