# Supplementary material for: Leprosy and the Adaptation of Human Toll-Like Receptor 1
Source: PLoS Pathog. 2010 Jul 1;6(7):e1000979. doi: 10.1371/journal.ppat.1000979 (PMC2895660; doi:10.1371/journal.ppat.1000979)
Supplement: Table S2 — Quality control criteria for the 50 K microarray genotyping for both samples and SNPs. (0.03 MB DOC) [file ppat.1000979.s010.doc]

|  |  | Odds ratio |  |
| --- | --- | --- | --- |
| Haplotype | Frequency | (ref=other haplotypes) | P-value |
| GTAGAC | 8.5% | 1.44 | 0.135 |
| CTAGAC | 13.0% | 1.21 | 0.343 |
| CCATAC | 17.2% | 0.88 | 0.487 |
| CTTTAC | 8.8% | 1.12 | 0.615 |
| CTTTCT | 8.7% | 0.29 | 1.3E-06 |
| CTTTAT | 42.9% | 1.24 | 0.117 |

**Table S11.** Haplotypic analysis of the *TLR 10/1/6* locus using SNPs rs7660429, rs11725309, rs10004195, rs7663239, rs5743618 (I602S) and rs4833095 in the New Delhi leprosy cohort.
