# Supplementary material for: Leprosy and the Adaptation of Human Toll-Like Receptor 1
Source: PLoS Pathog. 2010 Jul 1;6(7):e1000979. doi: 10.1371/journal.ppat.1000979 (PMC2895660; doi:10.1371/journal.ppat.1000979)
Supplement: Table S3 — Association statistics of all SNPs with P<1×10^−4^ in the Pearson's χ2 test in the primary association analysis in the New Delhi cohort. Statistically significant associations in the New Delhi cohort were verified with a logistic regression model, correcting for sex and age as covariates. (0.09 MB DOC) [file ppat.1000979.s011.doc]

| **Chr** | **Position** | **SNP** | **Gene** | **MAF case** | **MAF control** | ***P*-value** | **OR** | **95% CI** |
| --- | --- | --- | --- | --- | --- | --- | --- | --- |
| 1 | 2059541 | rs3753242 | *PRKCZ* | 24.9% | 13.6% | 1.7E-05 | 2.1 | 1.49-2.97 |
| 1 | 67567011 | rs3790562 | *IL12RB2* | 11.5% | 4.4% | 7.2E-05 | 2.82 | 1.66-4.8 |
| 1 | 85648210 | rs6673833 | *DDAH1* | 15.0% | 26.6% | 2.4E-05 | 0.49 | 0.35-0.68 |
| 1 | 204661692 | rs2244510 | *SRGAP2* | 27.0% | 40.2% | 3.5E-05 | 0.55 | 0.42-0.73 |
| 1 | 204667615 | rs9429893 | *SRGAP2* | 27.3% | 40.0% | 6.4E-05 | 0.56 | 0.42-0.75 |
| 1 | 204723200 | rs11118087 | *IKBKE* | 39.2% | 26.2% | 2.9E-05 | 1.82 | 1.37-2.42 |
| 2 | 164817572 | rs6707461 | *-* | 26.8% | 39.1% | 9.5E-05 | 0.57 | 0.43-0.76 |
| 3 | 10229797 | rs11465897 | *IRAK2* | 11.5% | 22.0% | 3.2E-05 | 0.46 | 0.32-0.67 |
| 3 | 128956720 | rs664910 | *MGLL* | 45.9% | 32.9% | 6.7E-05 | 1.73 | 1.32-2.28 |
| 3 | 128956957 | rs16830415 | *MGLL* | 21.4% | 10.4% | 6.8E-06 | 2.35 | 1.61-3.42 |
| 3 | 128964929 | rs547801 | *MGLL* | 24.2% | 11.8% | 1.2E-06 | 2.39 | 1.67-3.42 |
| 3 | 140696593 | rs295470 | *RBP1* | 58.2% | 42.3% | 2.0E-06 | 1.9 | 1.46-2.49 |
| 3 | 140719373 | rs2071388 | *RBP1* | 57.9% | 42.1% | 2.2E-06 | 1.9 | 1.45-2.47 |
| 4 | 38442115 | rs10008492 | *TLR10* | 2.9% | 11.3% | 1.6E-06 | 0.23 | 0.12-0.44 |
| 4 | 38475043 | rs5743618 | *TLR1* | 3.8% | 13.0% | 1.3E-06 | 0.27 | 0.15-0.47 |
| 4 | 110878516 | rs10033900 | *PLA2G12A* | 25.5% | 37.7% | 9.9E-05 | 0.57 | 0.42-0.75 |
| 6 | 21215088 | rs9358391 | *CDKAL1* | 39.5% | 26.8% | 5.3E-05 | 1.78 | 1.35-2.36 |
| 6 | 31484968 | rs12660741 | *MICA* | 18.8% | 9.7% | 9.9E-05 | 2.15 | 1.45-3.18 |
| 6 | 32253183 | rs3134947 | *RNF5* | 8.6% | 18.8% | 1.1E-05 | 0.41 | 0.27-0.61 |
| 6 | 32257794 | rs3134940 | *AGER* | 8.3% | 17.9% | 2.8E-05 | 0.41 | 0.27-0.63 |
| 6 | 32673832 | rs9270650 | *HLA-DRB1* | 43.8% | 24.3% | 6.4E-10 | 2.43 | 1.83-3.23 |
| 6 | 32677669 | rs477515 | *HLA-DRB1/DQA1* | 13.2% | 27.2% | 2.2E-07 | 0.41 | 0.29-0.57 |
| 6 | 32678378 | rs2516049 | *HLA-DRB1/DQA1* | 13.5% | 27.2% | 5.5E-07 | 0.42 | 0.29-0.59 |
| 6 | 32682038 | rs9270986 | *HLA-DRB1/DQA1* | 40.9% | 22.0% | 1.4E-09 | 2.45 | 1.83-3.29 |
| 6 | 32684042 | rs482044 | *HLA-DRB1/DQA1* | 34.7% | 47.7% | 9.3E-05 | 0.58 | 0.44-0.76 |
| 6 | 32710460 | rs3104369 | *HLA-DQA1* | 47.6% | 31.2% | 4.8E-07 | 2.01 | 1.53-2.64 |
| 6 | 32717104 | rs1071630 | *HLA-DQA1* | 31.4% | 52.2% | 8.5E-10 | 0.42 | 0.32-0.56 |
| 6 | 32734250 | rs9273363 | *HLA-DQA1* | 14.6% | 25.3% | 7.1E-05 | 0.50 | 0.36-0.71 |
| 6 | 32742445 | rs3891175 | *HLA-DQA1* | 11.2% | 22.8% | 9.6E-06 | 0.43 | 0.29-0.63 |
| 6 | 33650456 | rs210137 | *BAK1* | 42.8% | 29.9% | 5.9E-05 | 1.76 | 1.33-2.31 |
| 6 | 33650516 | rs210138 | *BAK1* | 43.7% | 30.2% | 3.2E-05 | 1.79 | 1.36-2.37 |
| 8 | 11714373 | rs7009163 | *FDFT1* | 6.7% | 17.2% | 3.4E-06 | 0.34 | 0.22-0.55 |
| 8 | 128812033 | rs16902359 | *MYC* | 39.0% | 25.6% | 1.9E-05 | 1.86 | 1.4-2.47 |
| 8 | 143996602 | rs1799998 | *CYP11B2* | 31.8% | 45.9% | 2.5E-05 | 0.55 | 0.42-0.73 |
| 9 | 34660128 | rs4879816 | *CCL27* | 9.1% | 19.0% | 2.3E-05 | 0.43 | 0.28-0.64 |
| 9 | 136453540 | rs3927491 | *RXRA* | 3.6% | 0.0% | 3.4E-05 | NA | NA |
| 10 | 32597598 | rs3740237 | *EPC1* | 33.3% | 21.1% | 5.2E-05 | 1.87 | 1.38-2.53 |
| 10 | 108697641 | rs17121799 | *SORCS1* | 15.1% | 6.9% | 8.7E-05 | 2.39 | 1.53-3.73 |
| 11 | 120903788 | rs666004 | *SORL1* | 42.5% | 55.9% | 6.7E-05 | 0.58 | 0.45-0.76 |
| 12 | 121781687 | rs525017 | *GPR81* | 25.1% | 14.0% | 2.6E-05 | 2.06 | 1.47-2.89 |
| 14 | 66899325 | rs8006042 | *EIF2S1* | 32.0% | 20.0% | 4.1E-05 | 1.89 | 1.39-2.56 |
| 14 | 66900609 | rs12588458 | *EIF2S1* | 36.8% | 23.7% | 2.4E-05 | 1.87 | 1.4-2.5 |
| 14 | 103236828 | rs861537 | *XRCC3* | 40.0% | 53.8% | 3.6E-05 | 0.57 | 0.44-0.75 |
| 15 | 46187491 | rs2675345 | *SLC12A1* | 29.0% | 16.3% | 5.7E-06 | 2.09 | 1.51-2.88 |
| 15 | 46282800 | rs1320052 | *SLC12A1* | 21.8% | 11.3% | 2.2E-05 | 2.19 | 1.52-3.15 |
| 15 | 46293975 | rs16960661 | *SLC12A1* | 20.2% | 9.2% | 3.1E-06 | 2.51 | 1.69-3.73 |
| 15 | 46301601 | rs9920281 | *SLC12A1* | 25.1% | 11.7% | 1.9E-07 | 2.53 | 1.77-3.61 |
| 15 | 46302144 | rs8032941 | *SLC12A1* | 12.0% | 4.6% | 4.8E-05 | 2.83 | 1.68-4.76 |
| 16 | 24042613 | rs1013316 | *PRKCB1* | 41.4% | 54.8% | 6.0E-05 | 0.58 | 0.45-0.76 |
| 17 | 19234320 | rs7501702 | *MFAP4* | 27.8% | 41.8% | 1.1E-05 | 0.53 | 0.4-0.71 |
| 19 | 51811873 | rs11083841 | *PTGIR* | 45.0% | 32.1% | 7.5E-05 | 1.73 | 1.32-2.27 |

**Table S3.** Association statistics of all SNPs with *P*<1x10^-4^ in the Pearson’s χ^2^ test in the primary association analysis in the New Delhi cohort. Statistically significant associations in the New Delhi cohort were verified with a logistic regression model, correcting for sex and age as covariates.
