# Supplementary material for: Leprosy and the Adaptation of Human Toll-Like Receptor 1
Source: PLoS Pathog. 2010 Jul 1;6(7):e1000979. doi: 10.1371/journal.ppat.1000979 (PMC2895660; doi:10.1371/journal.ppat.1000979)
Supplement: Table S4 — Association statistics of SNPs in the replication cohorts with susceptibility to leprosy. All SNPs with nominal P<0.05 with the same direction in either of the replication cohorts were highlighted in bold. (0.10 MB DOC) [file ppat.1000979.s012.doc]

|  |  |  |  |  | **Kolkata cohort** | | | **Kumbakonam cohort** | | |
| --- | --- | --- | --- | --- | --- | --- | --- | --- | --- | --- |
| **Chr** | **SNP** | **Gene** | **BP** | **Allele** | ***P*-value** | **OR** | **95% CI** | ***P*-value** | **OR** | **95% CI** |
| 1 | rs17883122 | *TNFRSF1B* | 12179528 | G | 0.660 | 1.38 | 0.33-5.85 | 0.683 | 0.85 | 0.38-1.89 |
| 1 | rs3790562 | *IL12RB2* | 67567011 | G | 0.102 | 0.62 | 0.35-1.1 | 0.325 | 1.19 | 0.84-1.70 |
| 1 | rs6673833 | *DDAH1* | 85648210 | G | 0.846 | 0.96 | 0.65-1.43 | 0.067 | 0.79 | 0.61-1.02 |
| 1 | rs2244510 | *SRGAP2* | 204661692 | C | 0.944 | 0.99 | 0.69-1.41 | 0.888 | 0.98 | 0.74-1.29 |
| 1 | rs9429893 | *SRGAP2* | 204667615 | G | 0.928 | 0.98 | 0.69-1.41 | 0.694 | 1.05 | 0.81-1.36 |
| 1 | rs11118087 | *IKBKE* | 204723200 | A | 0.528 | 1.12 | 0.79-1.57 | 0.506 | 1.08 | 0.87-1.34 |
| 3 | rs11465897 | *IRAK2* | 10229797 | A | 0.653 | 0.88 | 0.51-1.52 | 0.622 | 0.91 | 0.62-1.34 |
| 3 | rs664910 | *MGLL* | 128956720 | G | 0.917 | 0.98 | 0.71-1.37 | 0.788 | 0.96 | 0.74-1.26 |
| 3 | rs16830415 | *MGLL* | 128956957 | C | 0.834 | 1.05 | 0.67-1.65 | 0.572 | 0.92 | 0.70-1.22 |
| 3 | rs295470 | *NA* | 140696593 | G | 0.577 | 1.11 | 0.77-1.59 | 0.444 | 1.10 | 0.86-1.42 |
| 3 | rs2071388 | *RBP1* | 140719373 | G | 0.441 | 0.87 | 0.61-1.24 | 0.842 | 1.03 | 0.79-1.33 |
| 3 | rs2243126 | *IL12A* | 161192519 | A | 0.860 | 0.78 | 0.05-12.51 | 1.000 | 1.00 | 0.32-3.10 |
| **4** | **rs10008492** | ***TLR10*** | **38442115** | **C** | **0.047** | **0.50** | **0.25-1** | **1.000** | **1.00** | **0.61-1.63** |
| **4** | **rs5743618** | ***TLR1*** | **38475293** | **C** | **0.011** | **0.40** | **0.2-0.83** | **0.090** | **0.61** | **0.35-1.09** |
| 4 | rs10033900 | *PLA2G12A* | 110878516 | C | 0.424 | 0.86 | 0.6-1.24 | 0.460 | 1.10 | 0.86-1.39 |
| 6 | rs17662372 | *CDKAL1* | 21148356 | A | 0.447 | 2.35 | 0.24-22.74 | 1.000 | 1.00 | 0.06-15.99 |
| 6 | rs9358391 | *CDKAL1* | 21215088 | T | 0.968 | 1.01 | 0.72-1.41 | 0.851 | 1.02 | 0.80-1.31 |
| 6 | rs3093661 | *TNF* | 31651737 | A | 0.721 | 1.14 | 0.55-2.38 | 0.059 | 0.65 | 0.42-1.02 |
| 6 | rs3093662 | *TNF* | 31652168 | G | 0.719 | 1.12 | 0.6-2.11 | 0.072 | 0.69 | 0.47-1.04 |
| **6** | **rs3134947** | ***RNF5*** | **32253183** | **A** | **0.007** | **0.54** | **0.35-0.85** | **0.039** | **0.68** | **0.48-0.98** |
| 6 | rs3134940 | *AGER* | 32257794 | G | 0.279 | 0.71 | 0.38-1.33 | 0.100 | 0.69 | 0.45-1.08 |
| **6** | **rs9270650** | ***HLA-DRB1*** | **32673832** | **C** | **7.5E-04** | **1.83** | **1.28-2.59** | **0.030** | **1.30** | **1.03-1.65** |
| 6 | rs2516049 | *HLA-DRB1* | 32678378 | G | 0.142 | 0.63 | 0.33-1.17 | 0.131 | 0.78 | 0.56-1.08 |
| **6** | **rs9270986** | ***HLA-DRB1*** | **32682038** | **A** | **0.003** | **1.66** | **1.18-2.34** | **0.046** | **1.28** | **1.00-1.64** |
| **6** | **rs1071630** | ***HLA-DQA1*** | **32717104** | **T** | **1.1E-05** | **0.46** | **0.32-0.65** | **0.050** | **0.80** | **0.65-1.00** |
| 6 | rs210137 | *BAK1* | 33650456 | T | 0.594 | 1.10 | 0.77-1.56 | 0.671 | 1.05 | 0.83-1.34 |
| 6 | rs210138 | *BAK1* | 33650516 | G | 0.923 | 0.98 | 0.71-1.37 | 0.957 | 1.01 | 0.81-1.24 |
| 8 | rs17875660 | *IKBKB* | 42248724 | T | 0.871 | 1.03 | 0.7-1.52 | 0.751 | 1.04 | 0.81-1.34 |
| 8 | rs16902359 | *MYC* | 128812033 | T | 0.910 | 1.02 | 0.71-1.48 | 0.785 | 1.04 | 0.79-1.36 |
| 8 | rs1799998 | *CYP11B2* | 143996602 | C | 0.836 | 1.04 | 0.72-1.49 | 0.641 | 1.06 | 0.84-1.33 |
| **9** | **rs4879816** | ***CCL27*** | **34660128** | **A** | **0.211** | **1.42** | **0.82-2.46** | **7.7E-04** | **0.58** | **0.42-0.80** |
| 10 | rs3740237 | *EPC1* | 32597598 | C | 0.359 | 1.19 | 0.82-1.73 | 0.647 | 0.94 | 0.73-1.22 |
| 10 | rs2303886 | *ALOX5* | 45198272 | T | 0.521 | 1.36 | 0.53-3.51 | 0.782 | 0.86 | 0.29-2.55 |
| 10 | rs1051070 | *FAS* | 90764752 | T | 0.467 | 0.52 | 0.09-3.13 | 0.157 | 3.00 | 0.61-14.86 |
| 10 | rs17121799 | *SORCS1* | 108697641 | A | 0.195 | 0.73 | 0.45-1.18 | 0.795 | 0.96 | 0.68-1.34 |
| 11 | rs4252314 | *IL10RA* | 117373727 | G | 0.898 | 0.83 | 0.05-13.4 | 0.180 | 0.25 | 0.03-2.24 |
| 11 | rs666004 | *SORL1* | 120903788 | G | 0.035 | 1.42 | 1.03-1.98 | 0.865 | 1.02 | 0.82-1.27 |
| 12 | rs4149651 | *TNFRSF1A* | 6307650 | G | 0.525 | 1.57 | 0.39-6.33 | 0.251 | 1.71 | 0.67-4.35 |
| 12 | rs525017 | *GPR81* | 121781687 | A | 0.122 | 0.61 | 0.32-1.15 | 0.423 | 1.24 | 0.73-2.10 |
| 14 | rs8006042 | *EIF2S1* | 66899325 | G | 0.362 | 1.25 | 0.77-2.03 | 0.025 | 0.60 | 0.38-0.94 |
| 14 | rs12588458 | *EIF2S1* | 66900609 | G | 0.106 | 1.37 | 0.94-2 | 0.178 | 0.83 | 0.64-1.09 |
| 14 | rs861537 | *XRCC3* | 103236828 | A | 0.648 | 0.91 | 0.61-1.36 | 0.541 | 0.92 | 0.71-1.20 |
| 15 | rs2675345 | *NA* | 46187491 | G | 0.192 | 1.26 | 0.89-1.79 | 0.127 | 0.85 | 0.68-1.05 |
| 15 | rs1320052 | *SLC12A1* | 46282800 | C | 0.193 | 1.29 | 0.88-1.91 | 0.064 | 0.82 | 0.66-1.01 |
| 15 | rs16960661 | *SLC12A1* | 46293975 | C | 0.377 | 1.19 | 0.81-1.77 | 0.050 | 0.79 | 0.63-1.00 |
| 15 | rs9920281 | *SLC12A1* | 46301601 | G | 0.616 | 1.10 | 0.76-1.58 | 0.089 | 0.82 | 0.65-1.03 |
| 15 | rs8032941 | *SLC12A1* | 46302144 | C | 0.278 | 1.47 | 0.73-2.93 | 0.082 | 0.74 | 0.52-1.04 |
| **16** | **rs1013316** | ***PRKCB1*** | **24042613** | **A** | **0.039** | **0.70** | **0.49-0.98** | **0.046** | **1.25** | **1.00-1.55** |
| 17 | rs7501702 | *MFAP4* | 19234320 | A | 0.950 | 0.99 | 0.67-1.46 | 0.715 | 1.05 | 0.82-1.33 |
| 19 | rs11083841 | *PTGIR* | 51811873 | G | 0.850 | 1.04 | 0.73-1.48 | 0.066 | 1.23 | 0.99-1.53 |
| 20 | rs6021275 | *NFATC2* | 49588531 | G | 0.500 | 1.13 | 0.79-1.63 | 0.889 | 1.02 | 0.78-1.34 |

**Table S4.** Association statistics of SNPs in the replication cohorts with susceptibility to leprosy. All SNPs with nominal *P*<0.05 with the same direction in either of the replication cohorts were highlighted in bold.
