# Supplementary material for: Leprosy and the Adaptation of Human Toll-Like Receptor 1
Source: PLoS Pathog. 2010 Jul 1;6(7):e1000979. doi: 10.1371/journal.ppat.1000979 (PMC2895660; doi:10.1371/journal.ppat.1000979)
Supplement: Table S5 — Full genotypic counts and association statistics for SNPs rs1071630, rs9270650 and rs5743618 (I602S) under different genetic models. All the models in Pearson's χ2 represent 1-df test except the genotypic model which has 2 degrees of freedom. (0.06 MB DOC) [file ppat.1000979.s013.doc]

| ***HLA-DRB1/DQA1*** | |  |  |  |  |  |  |
| --- | --- | --- | --- | --- | --- | --- | --- |
| SNP | Genotype | Case | Control | Association statistics under different models | | | |
| rs1071630 | CC | 23 (11.6%) | 65 (28.3%) | OR (95% CI) | 0.42 (0.32-0.56) | *P* (dominant) | 8.2E-08 |
| New Delhi | TC | 79 (39.7%) | 110 (47.8%) | *P* (allelic) | 8.5E-10 | *P* (recessive) | 1.9E-05 |
|  | TT | 97 (48.7%) | 55 (23.9%) | *P* (trend) | 4.8E-09 | *P* (LR) | 1.2E-08 |
|  | Total | 199 | 230 | *P* (genotypic, 2df) | 3.0E-09 | *P* (LR | age + sex) | 2.1E-08 |
|  | *P* (HWE) | 0.25 | 0.51 | OR (LR | age + sex + MDS) | 2.15 (1.60-2.90) | *P* (LR | age + sex + MDS) | 4.0E-07 |
|  |  |  |  |  |  |  |  |
| SN*P* | Genotype | Case | Control | Association statistics under different models | | | |
| rs9270650 | TT | 66 (31.6%) | 136 (56.9%) | OR (95% CI) | 2.70 (1.93-3.76) | *P* (dominant) | 7.7E-08 |
| New Delhi | CT | 103 (49.3%) | 90 (37.7%) | *P* (allelic) | 6.4E-10 | *P* (recessive) | 7.5E-06 |
|  | CC | 40 (19.1%) | 13 (5.4%) | *P* (trend) | 1.2E-09 | *P* (LR) | 3.8E-09 |
|  | Total | 209 | 239 | *P* (genotypic, 2df) | 9.0E-09 | *P* (LR | age + sex) | 8.3E-09 |
|  | *P* (HWE) | 1.00 | 0.86 | OR (LR | age + sex + MDS) | 2.37 (1.72-3.25) | *P* (LR | age + sex + MDS) | 1.1E-07 |
|  |  |  |  |  |  |  |  |
| ***TLR1*** |  |  |  |  |  |  |  |
| SN*P* | Genotype | Case | Control | Association statistics under different models | | | |
| rs5743618 | AA | 193 (92.3%) | 184 (77%) | OR (95% CI) | 0.27 (0.15-0.47) | *P* (dominant) | 9.0E-06 |
| I602S | CA | 16 (7.7%) | 48 (20.1%) | *P* (allelic) | 1.3E-06 | *P* (recessive) | 0.013 |
| New Delhi | CC | 0 (0%) | 7 (2.9%) | *P* (trend) | 3.9E-06 | *P* (LR) | 1.5E-05 |
|  | Total | 209 | 239 | *P* (genotypic, 2df) | 2.4E-05 | *P* (LR | age + sex) | 2.1E-05 |
|  | *P* (HWE) | 1.00 | 0.08 | OR (LR | age + sex + MDS) | 0.35 (0.19-0.63) | *P* (LR | age + sex + MDS) | 4.8E-04 |
| rs5743618 | AA | 157 (93.4%) | 109 (83.2%) | OR (95% CI) | 0.40 (0.20-0.83) | *P* (dominant) | 0.005 |
| I602S | CA | 10 (6.0%) | 22 (16.8%) | *P* (allelic) | 0.012 | *P* (recessive) | 0.376 |
| Kolkata | CC | 1 (0.6%) | 0 (0%) | *P* (trend) | 0.012 | *P* (genotypic, 2df) | 0.008 |
|  | Total | 168 | 131 | *P* (HWE, case) | 0.078 | *P* (HWE, control) | 0.294 |

**Table S5.** Full genotypic counts and association statistics for SNPs rs1071630, rs9270650 and rs5743618 (I602S) under different genetic models. All the models in Pearson’s χ2 represent 1-df test except the genotypic model which has 2 degrees of freedom.
