# Supplementary material for: Leprosy and the Adaptation of Human Toll-Like Receptor 1
Source: PLoS Pathog. 2010 Jul 1;6(7):e1000979. doi: 10.1371/journal.ppat.1000979 (PMC2895660; doi:10.1371/journal.ppat.1000979)
Supplement: Table S6 — Stratified association analyses (leprosy type and age) and linear regression analysis (age of onset) for the three replicated SNPs rs1071630, rs9270650 and rs5743618 (I602S) at HLA-DRB1/DQA1 and TLR1. The Pearson's χ2 test was used for the stratified analyses, and the test of heterogeneity was performed with Woolf's test. (0.05 MB DOC) [file ppat.1000979.s014.doc]

|  |  |  | **Stratified analysis** | | **Stratified analysis** | |
| --- | --- | --- | --- | --- | --- | --- |
| **SNP** | **Allele** | **Statistics** | **Multibacillary** | **Paucibacillary** | **Age < 30** | **Age > 30** |
| rs1071630 | T | OR (95% CI) | 0.37 (0.26-0.53) | 0.48 (0.34-0.68) | 0.40 (0.28-0.59) | 0.45 (0.29-0.69) |
|  |  | *P*-value | 1.99E-08 | 2.99E-05 | 2.32E-06 | 2.65E-04 |
|  |  |  | Heterogeneity test *P*=0.33 | | Heterogeneity test *P*=0.72 | |
|  |  |  |  |  |  |  |
| rs9270650 | C | OR (95% CI) | 2.27 (1.61-3.20) | 2.61 (1.84-3.69) | 2.75 (1.88-4.03) | 1.95 (1.25-3.06) |
|  |  | *P*-value | 1.60E-06 | 3.75E-08 | 1.24E-07 | 0.003314 |
|  |  |  | Heterogeneity test *P*=0.58 | | Heterogeneity test *P*=0.25 | |
|  |  |  |  |  |  |  |
| rs5743618 | C | OR (95% CI) | 0.22 (0.10-0.50) | 0.31 (0.15-0.64) | 0.42 (0.19-0.95) | 0.16 (0.07-0.35) |
| I602S |  | *P*-value | 7.31E-05 | 9.06E-04 | 0.033 | 7.10E-07 |
|  |  |  | Heterogeneity test *P*=0.55 | | Heterogeneity test *P*=0.084 | |
|  |  |  |  |  | | |
|  |  |  | **Linear regression** |  | | |
| **SN*P*** | **Allele** | **Statistics** | **Age of onset** |  | | |
| rs1071630 | T | Beta | 0.79 |  | | |
|  |  | *P*-value | 0.53 |  | | |
|  |  |  |  |  | | |
| rs9270650 | C | Beta | 1.76 |  | | |
|  |  | *P*-value | 0.15 |  | | |
|  |  |  |  |  | | |
| rs5743618 | C | Beta | -2.76 |  | | |
| I602S |  | *P*-value | 0.39 |  | | |

**Table S6.** Stratified association analyses (leprosy type and age) and linear regression analysis (age of onset) for the three replicated SNPs rs1071630, rs9270650 and rs5743618 (I602S) at *HLA-DRB1/DQA1* and *TLR1*. The Pearson’s χ2 test was used for the stratified analyses, and the test of heterogeneity was performed with Woolf’s test.
