# Supplementary material for: Leprosy and the Adaptation of Human Toll-Like Receptor 1
Source: PLoS Pathog. 2010 Jul 1;6(7):e1000979. doi: 10.1371/journal.ppat.1000979 (PMC2895660; doi:10.1371/journal.ppat.1000979)
Supplement: Table S7 — Conditional logistic regression analysis of SNPs associated with leprosy susceptibility (P<0.05) at the HLA-DRB1/DQA1 locus. (0.04 MB DOC) [file ppat.1000979.s015.doc]

|  |  |  | **Allelic test** | | **Logistic regression** | | **LR SNP | rs9270650** | | **LR SNP | rs1071630** | | **LR SNP | rs9270650 + rs1071630** | | **LR SNP | rs477515** | |
| --- | --- | --- | --- | --- | --- | --- | --- | --- | --- | --- | --- | --- | --- | --- |
| CHR | SNP | BP | OR | P | OR | P | OR | P | OR | P | OR | P | OR | P |
| 6 | rs9270650 | 32673832 | 2.43 | 6.4E-10 | 2.46 | 3.8E-09 | NA | NA | 1.82 | 0.0014 | NA | NA | 2.08 | 5.1E-06 |
| 6 | rs477515 | 32677669 | 0.41 | 2.2E-07 | 0.42 | 1.3E-06 | 0.56 | 0.0021 | 0.62 | 0.0276 | 0.62 | 0.0316 | NA | NA |
| 6 | rs2516049 | 32678378 | 0.42 | 5.5E-07 | 0.43 | 2.7E-06 | 0.59 | 0.0060 | 0.63 | 0.0363 | 0.64 | 0.0442 | NA | NA |
| 6 | rs9270986 | 32682038 | 2.45 | 1.4E-09 | 2.40 | 1.3E-08 | 1.19 | 0.7105 | 1.74 | 0.0028 | 1.08 | 0.8701 | 2.04 | 9.4E-06 |
| 6 | rs482044 | 32684042 | 0.58 | 9.5E-05 | 0.61 | 0.0002 | 1.00 | 0.9776 | 0.61 | 0.0007 | 0.73 | 0.1212 | 0.86 | 0.3478 |
| 6 | rs3104369 | 32710460 | 2.01 | 4.8E-07 | 2.02 | 1.1E-06 | 1.02 | 0.9441 | 1.36 | 0.0960 | 0.82 | 0.4363 | 1.64 | 0.0015 |
| 6 | rs2040406 | 32710985 | 0.60 | 0.017 | 0.64 | 0.0272 | 0.86 | 0.4972 | 1.16 | 0.5428 | 1.17 | 0.5203 | 0.50 | 0.0011 |
| 6 | rs1071630 | 32717104 | 0.42 | 8.5E-10 | 0.44 | 1.2E-08 | 0.61 | 0.0041 | NA | NA | NA | NA | 0.54 | 0.0003 |
| 6 | rs9273363 | 32734250 | 0.50 | 7.1E-05 | 0.51 | 0.0001 | 0.72 | 0.0761 | 0.84 | 0.4422 | 0.86 | 0.5109 | 0.63 | 0.0114 |
| 6 | rs3891175 | 32742445 | 0.43 | 9.6E-06 | 0.46 | 5.0E-05 | 0.65 | 0.0341 | 0.88 | 0.6014 | 0.90 | 0.6516 | 0.60 | 0.0126 |

**Table S7.** Conditional logistic regression analysis of SNPs associated with leprosy susceptibility (*P*<0.05) at the *HLA-DRB1/DQA1* locus.
