# Supplementary material for: Leprosy and the Adaptation of Human Toll-Like Receptor 1
Source: PLoS Pathog. 2010 Jul 1;6(7):e1000979. doi: 10.1371/journal.ppat.1000979 (PMC2895660; doi:10.1371/journal.ppat.1000979)
Supplement: Table S8 — Bi-variate haplotypic analysis with SNPs rs9270650-rs1071630 in the HLA. The P-value statistics in each population were given by the likelihood ratio test in PLINK [40]. The combined values were given by the Mantel Haenszel statistics after confirming non-heterogeneity with Woolf's test of heterogeneity. (0.07 MB DOC) [file ppat.1000979.s016.doc]

| **rs9270650-rs1071630 haplotype** | | |  |  |  |  |  |
| --- | --- | --- | --- | --- | --- | --- | --- |
| **New Delhi** |  |  |  |  |  |  |  |
| Haplotype | Case Freq | Cont Freq | OR | L95 | U95 | *P*-value | |
| TC | 31.41% | 52.17% | 0.44 | 0.332 | 0.584 | 2.82E-09 |  |
| CT | 44.72% | 24.78% | 2.479 | 1.83 | 3.364 | 9.23E-10 |  |
| TT | 23.87% | 23.04% | 1.042 | 0.772 | 1.41 | 0.787 |  |
|  |  |  |  |  |  |  |  |
| **Kolkata** |  |  |  |  |  |  |  |
| Haplotype | Case Freq | Cont Freq | OR | L95 | U95 | *P*-value |  |
| TC | 27.41% | 48.03% | 0.412 | 0.28 | 0.605 | 2.31E-06 |  |
| CT | 49.26% | 34.50% | 1.683 | 1.2 | 2.36 | 0.002 |  |
| TT | 23.33% | 17.47% | 1.427 | 0.934 | 2.18 | 0.0958 |  |
|  |  |  |  |  |  |  |  |
| **Kumbakonam** |  |  |  |  |  |  |  |
| Haplotype | T | U | OR | L95 | U95 | *P*-value |  |
| TC | 134 | 155 | 0.8645 | 0.6861 | 1.089 | 0.2167 |  |
| CT | 139 | 109 | 1.275 | 0.9924 | 1.639 | 0.05678 |  |
| TT | 112 | 121 | 0.9256 | 0.7158 | 1.197 | 0.5555 |  |
|  |  |  |  |  |  |  |  |
| **Combined case-control statistics for haplotype TC** | | | | | | |  |
| *P*-value | 7.3x10^-15^ |  | OR | 0.41 |  |  |  |
| Heterogeneity *P* | 0.91 |  | 95% CI | 0.33-0.52 |  |  |  |
|  |  |  |  |  |  |  |  |
| **Combined case-control statistics for haplotype CT** | | | | | | |  |
| *P*-value | 5.2x10^-12^ |  | OR | 2.20 |  |  |  |
| Heterogeneity *P* | 0.23 |  | 95% CI | 1.75-2.76 |  |  |  |

**Table S8.** Bi-variate haplotypic analysis with SNPs rs9270650-rs1071630 in the *HLA*. The *P*-value statistics in each population were given by the likelihood ratio test in PLINK [40]. The combined values were given by the Mantel Haenszel statistics after confirming non-heterogeneity with Woolf’s test of heterogeneity.
