# Supplementary material for: Leprosy and the Adaptation of Human Toll-Like Receptor 1
Source: PLoS Pathog. 2010 Jul 1;6(7):e1000979. doi: 10.1371/journal.ppat.1000979 (PMC2895660; doi:10.1371/journal.ppat.1000979)
Supplement: Table S9 — Allelic dosage analysis for number of risk alleles at SNPs rs9270650 and rs1071630 on susceptibility to leprosy. (0.03 MB DOC) [file ppat.1000979.s017.doc]

| **Allelic dosage analysis** |  |  |  |  |  |  |
| --- | --- | --- | --- | --- | --- | --- |
| No. risk alleles | 0 | 1 | 2 | 3 | 4 | All |
| Control | 65 | 45 | 84 | 23 | 13 | 230 |
| Case | 23 | 25 | 67 | 44 | 40 | 199 |
| *Total* | 88 | 70 | 151 | 67 | 53 | 429 |
|  |  |  |  |  |  |  |
| Odds ratio (against others) | 0.24 | 0.39 | 1.13 | 1.69 | 3.01 |  |
| Odds ratio (against null allele) | 1 | 1.61 | 3.86 | 5.54 | 8.70 |  |
|  |  |  |  |  |  |  |
| Cochran-Armitage test *P* | 3.2x10^-11^ | |  |  |  |  |
| Logistic regression *P* | 1.1x10^-11^ | |  |  |  |  |

**Table S9.** Allelic dosage analysis for number of risk alleles at SNPs rs9270650 and rs1071630 on susceptibility to leprosy.
