# Supplementary material for: Leprosy and the Adaptation of Human Toll-Like Receptor 1
Source: PLoS Pathog. 2010 Jul 1;6(7):e1000979. doi: 10.1371/journal.ppat.1000979 (PMC2895660; doi:10.1371/journal.ppat.1000979)
Supplement: Table S10 — Conditional logistic regression analysis of SNPs associated with leprosy susceptibility (P<0.05) on the TLR1 I602S polymorphism. (0.03 MB DOC) [file ppat.1000979.s018.doc]

|  |  |  | **Allelic test** | | **Logistic regression** | | **LR SNP | I602S** | |
| --- | --- | --- | --- | --- | --- | --- | --- | --- |
| CHR | SNP | BP | OR | *P*-value | OR | *P*-value | OR | *P*-value |
| 4 | rs10008492 | 38442115 | 0.233 | 1.6E-06 | 0.247 | 1.9E-05 | 0.4996 | 0.1209 |
| 4 | rs4286521 | 38471701 | 0.331 | 0.0016 | 0.355 | 0.0042 | 1.471 | 0.4951 |
| 4 | rs5743618 | 38475043 | 0.267 | 1.2E-06 | 0.287 | 1.5E-05 | NA | NA |
| 4 | rs5743594 | 38479146 | 0.353 | 0.0021 | 0.375 | 0.0050 | 1.558 | 0.4267 |
| 4 | rs4833103 | 38491897 | 0.226 | 0.0368 | 0.222 | 0.0540 | 0.4348 | 0.3126 |
| 4 | rs2381289 | 38503266 | 0.759 | 0.0454 | 0.769 | 0.0520 | 0.891 | 0.4131 |
| 4 | rs5743818 | 38505558 | 0.429 | 0.0004 | 0.452 | 0.0009 | 0.9202 | 0.7923 |
| 4 | rs5743810 | 38506745 | 0.094 | 0.0047 | 0.091 | 0.0220 | 0.1536 | 0.0796 |

**Table S10.** Conditional logistic regression analysis of SNPs associated with leprosy susceptibility (*P*<0.05) on the *TLR1* I602S polymorphism.
