# Supplementary material for: Leprosy and the Adaptation of Human Toll-Like Receptor 1
Source: PLoS Pathog. 2010 Jul 1;6(7):e1000979. doi: 10.1371/journal.ppat.1000979 (PMC2895660; doi:10.1371/journal.ppat.1000979)
Supplement: Table S12 — Pairwise and global FST statistics of the 12 SNPs genotyped in the six populations recruited from New Delhi, Kolkata, Kumbakonam of India, Malawi, The Gambia and the United Kingdom. The SNP rs5743618 (TLR1 I602S) is highlighted in bold. (0.05 MB DOC) [file ppat.1000979.s020.doc]

| **Pairwise F_ST_** | rs6824105 | rs11096956 | rs4274855 | rs12233670 | **rs5743618** | rs5743613 | rs4833095 | rs5743595 | rs5743594 | rs2101521 | rs5743810 | rs5743795 |
| --- | --- | --- | --- | --- | --- | --- | --- | --- | --- | --- | --- | --- |
| Kolkata vs New Delhi | 0.00 | 0.00 | 0.00 | 0.01 | 0.01 | 0.00 | 0.00 | 0.00 | 0.01 | 0.00 | 0.00 | 0.00 |
| Kolkata vs Kumbakonam | 0.00 | 0.00 | 0.00 | 0.00 | 0.01 | 0.00 | 0.00 | 0.00 | 0.00 | 0.00 | 0.00 | 0.00 |
| Kolkata vs Malawi | 0.00 | 0.02 | 0.10 | 0.01 | 0.03 | 0.01 | 0.19 | 0.10 | 0.00 | 0.07 | 0.00 | 0.11 |
| Kolkata vs The Gambia | 0.00 | 0.00 | 0.09 | 0.01 | 0.02 | 0.00 | 0.16 | 0.08 | 0.02 | 0.08 | 0.00 | 0.10 |
| Kolkata vs UK | 0.07 | 0.01 | 0.00 | 0.03 | 0.35 | 0.00 | 0.05 | 0.00 | 0.05 | 0.02 | 0.26 | 0.00 |
| New Delhi vs Kumbakonam | 0.00 | 0.00 | 0.00 | 0.00 | 0.03 | 0.00 | 0.01 | 0.00 | 0.02 | 0.01 | 0.01 | 0.00 |
| New Delhi vs Malawi | 0.00 | 0.04 | 0.10 | 0.03 | 0.05 | 0.01 | 0.24 | 0.09 | 0.02 | 0.11 | 0.01 | 0.10 |
| New Delhi vs The Gambia | 0.01 | 0.00 | 0.09 | 0.04 | 0.05 | 0.00 | 0.21 | 0.08 | 0.04 | 0.11 | 0.01 | 0.09 |
| New Delhi vs UK | 0.05 | 0.02 | 0.00 | 0.01 | 0.29 | 0.00 | 0.02 | 0.00 | 0.02 | 0.00 | 0.25 | 0.00 |
| Kumbakonam vs Malawi | 0.00 | 0.04 | 0.09 | 0.01 | 0.01 | 0.01 | 0.18 | 0.09 | 0.00 | 0.05 | 0.00 | 0.11 |
| Kumbakonam vs The Gambia | 0.00 | 0.00 | 0.08 | 0.02 | 0.00 | 0.00 | 0.15 | 0.08 | 0.01 | 0.06 | 0.00 | 0.10 |
| Kumbakonam vs UK | 0.08 | 0.02 | 0.00 | 0.02 | 0.41 | 0.00 | 0.05 | 0.00 | 0.07 | 0.03 | 0.28 | 0.00 |
| Malawi vs The Gambia | 0.00 | 0.02 | 0.00 | 0.00 | 0.00 | 0.00 | 0.00 | 0.00 | 0.01 | 0.00 | 0.00 | 0.00 |
| Malawi vs UK | 0.09 | 0.00 | 0.10 | 0.07 | 0.46 | 0.00 | 0.39 | 0.11 | 0.07 | 0.15 | 0.28 | 0.10 |
| The Gambia vs UK | 0.09 | 0.01 | 0.09 | 0.08 | 0.45 | 0.00 | 0.35 | 0.10 | 0.09 | 0.16 | 0.28 | 0.10 |
|  |  |  |  |  |  |  |  |  |  |  |  |  |
| **Global F_ST_** | 0.04 | 0.02 | 0.06 | 0.04 | 0.39 | 0.01 | 0.21 | 0.06 | 0.07 | 0.10 | 0.35 | 0.07 |

**Table S12.** Pairwise and global F_ST_ statistics of the 12 SNPs genotyped in the six populations recruited from New Delhi, Kolkata, Kumbakonam of India, Malawi, The Gambia and the United Kingdom. The SNP rs5743618 (*TLR1* I602S) is highlighted in bold.
