# Supplementary material for: Leprosy and the Adaptation of Human Toll-Like Receptor 1
Source: PLoS Pathog. 2010 Jul 1;6(7):e1000979. doi: 10.1371/journal.ppat.1000979 (PMC2895660; doi:10.1371/journal.ppat.1000979)
Supplement: Table S13 — Association statistics of rs5743618 (TLR1 I602S) in a Gambian tuberculosis case control population. (0.03 MB DOC) [file ppat.1000979.s021.doc]

| ***TLR1 I602S*** | |  |  |
| --- | --- | --- | --- |
| SNP | Genotype | Case | Control |
| rs5743618 | AA | 327 (95.6%) | 337 (96.6%) |
| (I602S) | AC | 15 (4.4%) | 12 (3.4%) |
|  | CC | 0 (0%) | 0 (0%) |
|  | Total | 342 | 337 |
|  | *P* (HWE) | 0.68 | 0.74 |
|  | *P* (allelic) | 0.523 |  |
|  | OR (95% CI) | 1.28 (0.60-2.76) |  |

**Table S13.** Association statistics of rs5743618 (*TLR1* I602S) in a Gambian tuberculosis case control population.
